# Chemical Profile and Antibacterial Activity of *Vitis vinifera* L. cv Graciano Pomace Extracts Obtained by Green Supercritical CO_2_ Extraction Method Against Multidrug-Resistant *Escherichia coli* Strains

**DOI:** 10.3390/foods14010017

**Published:** 2024-12-25

**Authors:** Rocío Fernández-Pérez, Silvia Ayuso, Cristina Moreta, María-José Saiz-Abajo, Miguel Gastón-Lorente, Fernanda Ruiz-Larrea, Carmen Tenorio

**Affiliations:** 1 Instituto de Ciencias de la Vid y del Vino (ICVV) (Universidad de La Rioja, Consejo Superior de Investigaciones Científicas (CSIC), Gobierno de La Rioja), 26007 Logroño, Spain; 2 National Centre for Food Technology and Safety (CNTA), 31570 San Adrian, Spain

**Keywords:** grape pomace, *Vitis vinifera*, polyphenols, supercritical fluid extraction, *Escherichia coli*, antibiotic resistance, antibacterial activity

## Abstract

The objectives of this study were to obtain and characterise polyphenolic extracts from red grape pomace of *Vitis vinifera* L. cv Graciano via conventional solvent extraction (SE) and green supercritical fluid extraction (SFE) and to evaluate their *in vitro* antibacterial activity against susceptible and multidrug-resistant *Escherichia coli* strains of intestinal origin. The SE and SFE methods were optimised, and ultra-performance liquid chromatography/mass spectrometry (UPLC/QqQ-MS/MS) analysis revealed 38 phenolic compounds in the SE sample, with anthocyanins being the predominant polyphenols, and 21 phenolic compounds in the SFE samples, among which hydroxybenzoic acids and flavonols were the predominant compounds. The SE and SFE samples showed antibacterial activity against both antibiotic-susceptible and -resistant *E. coli* strains, and minimal inhibitory concentration values were in the range of 1–4 mg/mL. The activity was bacteriostatic in all cases, and it was shown that a higher content of total polyphenols correlated with a higher antibacterial activity of the extracts. This study shows that red grape pomace of *Vitis vinifera* L. cv. Graciano is a rich source of bioactive phenolic compounds that can become an important feedstock for additives and other upgraded products of biotechnological interest, which can help to modulate intestinal microbiota and combat bacterial antibiotic resistance.

## 1. Introduction

The winemaking process generates great amounts of grape pomace, whose efficient exploitation is currently a challenge in the context of the circular economy, and which may become a suitable candidate for developing viable, sustainable, eco-friendly and bio-based products. Grape pomace, which is made up mainly of skins and seeds, is estimated to represent around 20–30% of the original grape weight used for the winemaking [1], and approximately 1 kg of grapes is required to produce 0.75 L of wine [2]. According to the International Organisation of Vine and Wine [3], 237 million hectolitres of wine was the global wine production in 2023. Taking into account these figures and 25% as the midway point in the estimated range (the aforementioned 20–30%) of the proportion of grape weight composed of pomace, 7.9 million tons was the world production of grape pomace last year. Grape pomace can be used to obtain ethanol or grape seed oil; nevertheless, substantial amounts of phenolic compounds remain in the waste product.

Phenolic compounds are secondary metabolites synthetised by plants to fight against environmental stress factors [4] such as UV radiation, temperature shifts, water deficits or infective and pathogenic agents. This cell protection function against various abiotic and biotic stressors has been mainly associated with the antioxidant activity of phenolic compounds and their interaction with reactive oxygen species [5]. This activity has been associated with a number of benefits for human health, and currently, phenolic compounds are used as therapeutic agents and nutritional supplements (see references [5,6] for recent reviews of plant polyphenols). The diversity of plant phenolic compounds, also referred to as polyphenols, is great, and they can be broadly divided into four classes: phenolic acids, flavonoids, stilbenes and lignans [7]. Phenolic acids include hydroxybenzoic and hydroxycinnamic acids. Flavonoids can be subdivided according to their degree of unsaturation and oxidation into subclasses, among which flavanols, flavones (which include flavonols and other flavones) and anthocyanins are included. Stilbenes contain two phenyl rings connected by a two-carbon ethylene bridge, and lignans contain two phenyl rings in a 2,3-diphenylbutane structure [7].

Novel extraction techniques of polyphenols from grape pomace have been the subject of considerable interest among researchers, and recent reviews can be found on emerging technologies for the extraction of polyphenols from grape pomace [8,9]. Conventional extraction from solid material is based on the use of solvents to macerate the material and achieve the mass transfer and solubilisation of the compounds of interest, whereas green extraction techniques need less solvent and avoid the use of organic solvents. Among green extraction techniques, the extraction based on supercritical carbon dioxide (CO_2_) is regarded as an environmentally friendly extraction method as it does not utilise organic solvents and no solvent residues are generated. CO_2_ is subjected to high pressures and pumped into the extraction chamber filled with the plant material. The pressurised CO_2_ possesses characteristic fluid properties, such as the absence of surface tension and very low viscosity, which enhance its penetrability into the small pores of the solid material, favouring the extraction of compounds, especially those with some hydrophobic character [10]. This supercritical fluid extraction (SFE) has been applied to red grape pomace, mainly of the *Vitis vinifera* Merlot variety (reviewed in [8,9,11,12,13]), and also to the pomace of the following varieties: Garnacha [14], Petit Verdot [15], Cabernet Sauvignon [16] and *Vitis labrusca* [17], which do not include Graciano. These previous studies focused mainly on the phenolic content, composition and antioxidant activity of the SFE extracts.

The Graciano grape is a *Vitis vinifera* red variety autochthonous of the Spanish Northern region of La Rioja [18]. It is officially named by a number of synonyms, and thus, it is known as Morrastrel in France, Cagnulari in Italy, Tinta Miúda in Portugal and Xeres in California [19]. According to the International Organisation of Vine and Wine, the Graciano variety is not included among the ten most widely cultivated grape varieties. Currently, it is mainly grown in Spain (2080 ha, in La Rioja and Navarra), followed by Italy (437 ha), Portugal (326 ha) and a few other countries that dedicate less than 50 ha to this cultivar [20]. It is worth noting that its cultivation is currently extending [21] due to its drought resistance, which is an essential trait to withstand the current global warming.

*Escherichia coli* is a relevant Gram-negative species of saprophytes of the gut microbiota of humans and animals; nevertheless, it may become an opportunistic pathogen, mainly when the host’s immune defence is impaired, and it may develop problematic antibiotic resistances that hinder treatments against bacterial infections. *E. coli* is also a common indicator of faecal contamination, antibiotic resistance and dissemination through the food chain [22]. Global dissemination of bacterial resistance to antibiotics is currently an issue of major concern. This problem is due to bacteria’s natural ability to mutate rapidly and acquire new antibiotic resistance genes, adding them to their own genome, and thus evading the effects of antibiotics. The spread of bacterial resistance was driven by the misuse of antibiotics [23]. Some of the major current measures for tackling this problem are the prudent use of antibiotics and the development of alternative antimicrobials that do not elicit antibiotic resistance. Aqueous soluble polyphenols purified from a variety of plants have been shown to possess antimicrobial activities, among which resveratrol and caffeic acid were found [6], and red wine was the reported natural source for these two bioactives. As mentioned above, red grape pomaces constitute a rich source of polyphenolic bioactive compounds, and they are current targets for valorisation as raw materials for value-added products [24]. Nevertheless, to our knowledge, to date, no study has been reported on the activity of SFE samples from grape pomace, fresh skins or seeds on the growth of antibiotic-resistant *E. coli* strains. A previous study of our research group reported antimicrobial activity of polyphenolic extracts from fresh red grape skins and seeds of *Vitis vinifera* of the Graciano variety against multidrug-resistant *E. coli* strains [25]. The objectives of this work were to obtain and characterise polyphenolic extracts from Graciano red grape pomace via conventional solvent extraction and green supercritical CO_2_ extraction using ethanol as co-solvent and to evaluate their *in vitro* antibacterial activity against susceptible and multidrug-resistant *E. coli* strains of animal intestinal origin.

## 2. Materials and Methods

### 2.1. Chemicals and Reagents

Standards for phenolic compounds were as follows. Caftaric acid (99.5%), *trans*-piceid (98%), cyanidin-3-O-glucoside chloride (99%), procyanidin B1 (99.5%), procyanidin B2 (98%), kaempferol-3-O-glucoside (99%), isorhamnetin (99.1%), myricetin (99.7%), taxifolin 3-O-rhamnoside (99.8%), procyanidin trimer C1 (98.4%) and quercetin-3-O-glucuronide (98%) were from Biopurify Phytochemicals (Chengdu, China). Malvidin-3-O-glucoside chloride, grade for high-performance liquid chromatography (HPLC ≥ 95%), peonidin-3-O-glucoside chloride (HPLC ≥ 95%), petunidin-3-O-glucoside chloride (HPLC ≥ 95%), delphinidin-3-O-glucoside chloride (HPLC ≥ 95%), isorhamnetin-3-O-glucoside (HPLC ≥ 98%), myricetin-3-O-galactoside (97%), luteolin-7-O-glucoside (97%), procyanidin dimer A2 (92%) and syringetin-3-O-glucoside (HPLC ≥ 99%) were from Extrasynthese (Genay, France). Matairesinol (HPLC ≥ 85%), secoisolariciresinol (HPLC ≥ 95%), caffeic acid (98%), *trans*-coumaric acid (98%), *trans*-ferulic acid (99%), gallic acid (97.5%), 4-hydroxybenzoic acid (99%), 4-hydroxyphenylacetic acid (98%), protocatechuic acid (97%), syringic acid (95%), vanillic acid (97%), *trans*-resveratrol (99.5%), catechin (98%), epicatechin (95%) and quercetin (97%) were from Sigma-Aldrich (St. Louis, MO, USA). Naringenin (95%), coumaric acid ethyl ester (97%), ferulic acid ethyl ester (99.87%) and phlorizin (98%) were from Fluorochem (Hadfield, UK). Kaempferol (98.3%) and apigenin (98.3%) were from Glentham Life Science (Corsham, UK). Caffeic acid ethyl ester (92.9%), apigenin-7-O-glucoside (99.79%) and gallocatechin (99.09%) were from Targetmol Chemicals (Boston, MA, USA). Tyrosol (98%), protocatechuic acid ethyl ester (98.8%) and 3-O-caffeoylquinic acid (99.58%) were from Apollo Scientific (Bredbury, UK). *Trans*-coutaric acid (97%) and *trans*-fertaric acid (90%) were from PhytoLab (Vestenbergsgreuth, Germany). Luteolin (98%) was from biosynth-carbosynth (Bratislava, Slovakia), phloretin (99.8%) was from TCI (Tokyo Chemical Industry) (Tokyo, Japan) and hydroxytyrosol (99.5%) was from Seprox Biotech (Murcia, Spain). Most of the compounds were acquired through the intermediary Cymit Química (Barcelona, Spain).

Regarding chemical reagents needed for the relevant analytical analyses (extraction and chemical analysis), acetonitrile high-performance liquid chromatography/mass spectrometry (HPLC/MS) grade, methanol HPLC/MS grade and formic acid HPLC/MS grade (99%) were purchased from Carlo Erba Reagents S.A.S. (Val-de-Reuil, France). DPPH (Sigma-Aldrich) (2,2-diphenyl-1-picrylhydrazyl) and Trolox (Sigma-Aldrich) (6-hydroxy-2,5,7,8-tetramethylchroman-2-carboxylic acid; 97%) were used for antioxidant activity determination. Ethanol 96% (VWR Int., Barcelona, Spain) and CO_2_ (Air Liquide Co., Madrid, Spain) were used for SFE. A Milli-Q^®^ Integral device equipped with an LC-Pak Cartridge attached to Millipak^®^ Express 40 final filter of 0.22 μm (Merck Millipore, Merck KGaA, Darmstadt, Germany) was employed to obtain ultrapure water for UPLC-MS.

### 2.2. Grape Pomace

The raw material was red grape pomace that was kindly provided by one winery located in the Northern Spanish region of Rioja. It was collected after traditional vinification of red *Vitis vinifera* L. cv. Graciano grapes during vintage 2021 and comprised mainly grape skins and seeds. A reduced number of short stems always remain attached to some of the grapes after the initial process of crushing and destemming the grapes from the stalks. Red grape pomace was frozen and stored at −20 °C until it was used for extraction procedures.

### 2.3. Total Phenolic Content (TPC)

The Folin–Ciocalteu assay [26] was performed to calculate the TPC of extraction samples. Briefly, a 100 μL properly diluted extract or a standard sample of varying concentration was dissolved into 1.4 mL of deionised water, and 2.5 mL Folin–Ciocalteu solution (1:10 *v*/*v* dilution in methanol) and 1 mL of carbonate solution (20% *w*/*v*) was added. The mixture was thoroughly mixed and kept in the dark for 90 min. The absorbance was measured at 725 nm in a spectrophotometer (730 UV–Visible spectrophotometer Jasco, Madrid, Spain) and compared with a calibration curve prepared with known concentrations of gallic acid, which was used as the standard to quantify the TPC of samples. The calibration curve was prepared with a stock solution of gallic acid in methanol, and standard samples were prepared in the range 0–750 μg/mL. Results were expressed as µg of equivalent gallic acid (GAE) per g of dried sample (µg_GAE_/g_DW_).

### 2.4. Antioxidant Activity (AA)

The DPPH assay [27] was used to determine AA. The stock solution of 2,2-diphenyl-2-picrylhydrazyl (DPPH) was 0.05 g/L of methanol. This stock solution was further diluted 1:5 in methanol to reach an absorbance of 0.75 ± 0.05 at 515 nm (Jasco 730 UV-Vis spectrophotometer) on the day of the analysis. Briefly, triplicates were prepared combining a 40 μL properly diluted extract or standard sample of varying concentrations with 1960 μL of the DPPH working solution, they were incubated for 1 h at room temperature in darkness, and absorbance was measured at 515 nm. A 100 µM stock solution of Trolox (6-hydroxy-2,5,7,8-tetramethylchromane-2-carboxylic acid) was used as standard to prepare the calibration curve in the range 5–60 µM and quantify AA. Results were expressed as mg of equivalent Trolox per g of dried sample (mg_Trolox_/g_DW_).

### 2.5. Conventional SE

The first procedure before extraction was grinding the frozen pomace in a Grindomix GM 200 knife mill (Retsch GmbH, Düsseldorf, Germany) until a homogenous mixture was obtained. The initial moisture content of the sample was 57.97 ± 0.93%.

Conditions for conventional SE with an acid hydroalcoholic solution of ethanol and 0.01% HCl were optimised following the Box–Behnken design method using the Nemrod-W^®^ software (version 2007, LPRAI, Marseille, France). This design leads to the optimisation of and the reduction in the number of experiments, and it also considers the possible interactions between the studied factors and their effects on the results. Following previous observations and reported SE methods in the bibliography, three independent variables and their corresponding value ranges were chosen to study the effects on TPC and AA of extracts: ethanol concentration, temperature and extraction time. The software generated an experimental design of 27 assays within the following design space: from 30% to 70% ethanol; from 30 °C to 50 °C; and from 1 h to 5 h extraction time (Table 1). Briefly, 3 g of the ground and defrosted pomace was weighed and mixed with 30 mL of the appropriate acid hydroalcoholic solution in a glass flask. The mixture was vortexed until homogeneity and incubated in a water bath under the established conditions of temperature and time of incubation. Afterwards, incubation samples were spun at 2425× *g* for 10 min. Finally, supernatants were submitted to TPC and AA analyses.

The resulting optimised conditions for SE (55% ethanol, 45 °C, 5 h) were used to obtain 250 mL of polyphenolic extract, which was freeze-dried (Telstar LyoAlfa 6-80 freeze-dryer, Barcelona, Spain) to obtain the SE sample named Es, which was weighed, assayed for TPC and AA and chemically analysed, as indicated in Section 2.3, Section 2.4 and Section 2.7.

### 2.6. SFE

A SFE pilot plant (SFF model, IberFluid/PID Eng and Tech. Madrid, Spain) equipped with an extraction vessel, two separators in series and a storage tank for CO_2_ (≥99.9% pure CO_2_) was used for supercritical CO_2_ extraction using ethanol as the co-solvent. Ethanol was chosen as co-solvent to increase the supercritical CO_2_ ability to dissolve polyphenols. Ethanol polarity is higher than that of CO_2_, and it increases the fluid solvating power in SFE [9]. Water was not included as co-solvent, as previous reports indicated that pectic substances accompanied phenolic compounds in the extract when water was included as co-solvent; moreover, slightly higher temperatures were required for the recovery of polyphenols when compared with ethanol as co-solvent [28].

Prior to SFE, the frozen grape pomace was freeze-dried and then ground in the Grindomix knife mill. Particles were classified according to particle size using a digital sieve shaker (RP200N model, CISA, Barcelona, Spain) with the following mesh sizes: 1250 μm, 1000 μm, 500 μm and 200 μm of particle diameter. Separations were performed for 3 min at 3000 vibrations per minute, 2.3 mm of vibration amplitude, in a discontinuous mode (intervals: 7.5 s on/2.5 s off).

Two variables were chosen for SFE: extraction total pressure and particle size. An experimental design, shown in Table 2, was generated within the following design space: from 150 to 600 bar and with two particle sizes.

The extraction vessel was filled with 50 g of the ground and sifted sample, and glass beads (mean diameter of 5 mm) were added. Supercritical CO_2_ extractions were carried out at the following fixed parameters: CO_2_ flow rate (2000 mL/h), ethanol concentration (10%), extraction time (3 h) and extraction temperature (40 °C). Samples were recovered in the three types of assays (SF1, SF2, SF3) from each of the three vessels: extractor (samples SF1.1, SF2.1, SF3.1), separator A (samples SF1.2, SF2.2, SF3.2) and separator B (samples SF1.3, SF2.3, SF3.3). After removal of the co-solvent in a rotary evaporator, the samples were weighed, analysed for TPC and AA and selected for subsequent antimicrobial and chemical analyses.

### 2.7. Chemical Analysis of the Extracts by UPLC/QqQ-MS/MS

Chemical analysis of the obtained polyphenolic extracts was performed by the Service of Instrumental Analysis of the Institute of Science of Vine and Wine (ICVV, Logroño, Spain). Phenolic profiles of the obtained extracts were identified by UPLC/QqQ-MS/MS using a liquid chromatograph (Shimadzu Nexera, Shimadzu Corporation, Kyoto, Japan) coupled with a QTRAP mass spectrometer (AB Sciex 3200QTRAP^®^, Sciex, Framingham, MA, USA) equipped with an ESI electrospray ionisation source (ESI Turbo V™ Source). The chromatographic separation was performed on a Waters Acquity UPLC BEH C18 column (100 mm × 2.1 mm, 1.7 μm particle size; Waters, Milford, MA, USA) equipped with a VanGuardTM AcQuity BEH C18 Pre-Column (5 mm × 2.1 mm, 1.7 μm particle size; Waters, Milford, MA, USA).

Two chromatographic methods were employed, one for the analysis of anthocyanins and another for the analysis of the rest of the phenolic compounds. In both methods, the flow rate was 0.45 mL/min, the injection volume was 2.5 μL and the autosampler and oven temperatures were 8 and 40 °C, respectively. The mobile phases were water (solvent A) and acetonitrile (solvent B) acidified with a 2% (*v*/*v*) of formic acid to separate anthocyanins and acidified with 0.1% (*v*/*v*) formic acid to separate the rest of the analytes. In both methods, the chromatographic separation took place in less than 15 min. The gradient elution program was as follows: 0–0.5 min, 1% B; 0.5–1.5 min, 1–8% B; 1.5–4.0 min, 8% B; 4.0–5.0 min, 8–12% B; 5.0–5.5 min, 12% B; 5.5–6.0 min, 12–14% B; 6.0–7.0 min, 14% B; 7.0–9.0 min, 14–22% B; 9.0–12.0 min, 22–30% B; 12.0–13.7 min, 30–50% B; 13.7–14.0 min, 50–90% B; 14.0–15.0 min, 90% B; and finally, returning to the initial conditions in 0.5 min and maintained 3 min before the next injection.

The eluted compounds were analysed by using a triple-quadrupole mass spectrometer. The electrospray interface was set in positive ion mode (ESI+) with an ion spray voltage of +4.5 kV for the analysis of the anthocyanins and in negative ion mode (ESI−) with an ion spray voltage of −3.5 kV for the analysis of the rest of the analytes. Nitrogen (degasified liquid nitrogen > 99.99% purity, Air Liquide, Madrid, Spain) was used as the source and collision gas. The source temperature was 700 °C, and the gas pressures were curtain gas 50 psi, GS1 50 psi and GS2 60 psi. The data were acquired by multiple reaction monitoring (MRM). The dwell time established for each transition (and number of data points across the UPLC peak) was optimised through the chromatogram employing the Scheduled MRM™ Algorithm by means of the retention time with an MRM detection window of 60 s and a target scan time of 1.2 s. The retention time and MRM transitions for quantification and identification, in addition to the individual declustering potential (DP), entrance potential (EP), collision cell entrance potential (CEP), collision energy (CE) and collision cell exit potential (CXP) for each phenolic compound are shown in Supplementary Table S1. Some of the phenolic compounds were identified and quantified using pure commercial standards. Analytes with no available standards were identified by studying and comparing MS fragmentation patterns and relative retention times described in the literature. Those analytes were tentatively quantified using the calibration curves of standards with similar chemical structures. The standard selected for each analyte is also shown in Supplementary Table S1.

Samples were prepared by diluting approximately 10 mg of each extract in 800 μL of MeOH/water/formic acid (60:39:1 *v*/*v*/*v*). When turbidity was observed, the sample was sonicated for 5–10 min in an ice-cold ultrasonic bath (temperature less than 20 °C). Samples were centrifuged at 4500× *g* (10 min, 4 °C) and filtered through a 0.22 μm PTFE filter (Scharlab, Catalonia, Spain) before the chromatographic analysis. Due to the wide range of concentrations of phenolic compounds in samples, a 1:10 dilution with MeOH/water/formic acid (20:79:1 *v*/*v*/*v*) was also injected. The final concentrations of phenolic compounds in each sample were determined by averaging the content after three consecutive injections, and the results are expressed as ng/mg of dry extract.

Data processing was performed with the software package Analyst 1.6.2 and MultiQuant^TM^ 3.0.2 (AB Sciex, Framingham, MA, USA).

### 2.8. Microbial Culture Conditions and Antimicrobial Assays

Four *E. coli* strains (Table 3) of animal intestinal origin were obtained from previous studies performed by the OneHealth-UR research group. Two strains were susceptible to antibiotics, and the other two were extended-spectrum beta-lactamase (ESBL)-producing strains and showed resistance to at least two families of antibiotics.

Strains were tested by the microtiter dilution method [29] to evaluate the antimicrobial activity of the polyphenolic extracts of our study: Es, SF1.3, SF2.3 and SF3.3. Cultures were performed in brain heart infusion (BHI) broth (Condalab, Madrid, Spain) in 96-well microdilution plates, and the multimode microplate reader Tecan Spark 10M (Tecan Trading AG, Männedorf, Switzerland) was used to measure bacterial growth by optical density at 600 nm. All assays were performed in triplicate, and negative and positive control samples were included in all the assays. Negative controls without bacterial inoculum were used to verify that there was no cross-contamination. Positive controls of bacterial growth in the absence of polyphenolic extract were used to verify the bacterial inoculum viability and its adequate cell growth in each assay. The optical density values of positive controls were the reference to determine bacterial growth inhibition in each assay. Consecutive 2-fold dilutions of each extract were prepared across each row of the microdilution plate. A total volume of 100 μL in each well contained 25 μL inoculum of the bacterial suspension (10^5^ cells/mL), 25 μL of extract sample with the appropriate serial dilution and BHI broth. Incubations were performed at 30 °C for 10–24 h. Controls were included in all the assays. MIC was defined as the lowest concentration of the extract that inhibited bacterial growth after 24 h incubation. Bactericide activity was determined by subculturing onto BHI agar plates without the tested extract for 36 h, and MBC was defined as the minimal concentration of the antimicrobial agent that had killed more than 99.9% of the initial inoculum after 48 h incubation onto BHI agar plates. The tested concentration range for the extracts was 8.0–0.13 mg/mL (double dilutions), and all the bacterial assays were performed in triplicate.

### 2.9. Statistical Analysis

Mean values and standard deviations of three repeats were calculated. Analysis of variance (ANOVA) was applied as the data showed normal distribution and homogeneous variances. Tukey’s range test was used as the mean comparison method of data obtained in all the performed physicochemical analyses at a significance level of *p* = 0.05. The IBM-SPSS Statistics 22.0 for Windows (IBM-SPSS Inc., Chicago, IL, USA) statistical package was used for data processing.

## 3. Results and Discussion

Pomace of *Vitis vinifera* red grapes of the Graciano variety was chosen for this study because this variety stands out for its high content of phenolic compounds [30], and these molecules are well known for playing a relevant role in plant stress response to a variety of external harassments such as drought, as mentioned above. This high content of phenolic compounds explains why the Graciano variety is regarded as a drought-tolerant red grapevine variety, as mentioned, and a candidate to attenuate the deleterious effects of climate change on grapevines. Regarding Graciano varietal red wines, their content of flavonoid compounds has been reported to be among the highest [31]. The starting material of Graciano red grape pomace in our study showed indeed a high TPC (68,220 ± 3190 mg_GAE_/g_DW_). This pomace was submitted to both the SE and SFE extraction methods described above.

### 3.1. Conventional SE

**Figure 1:**
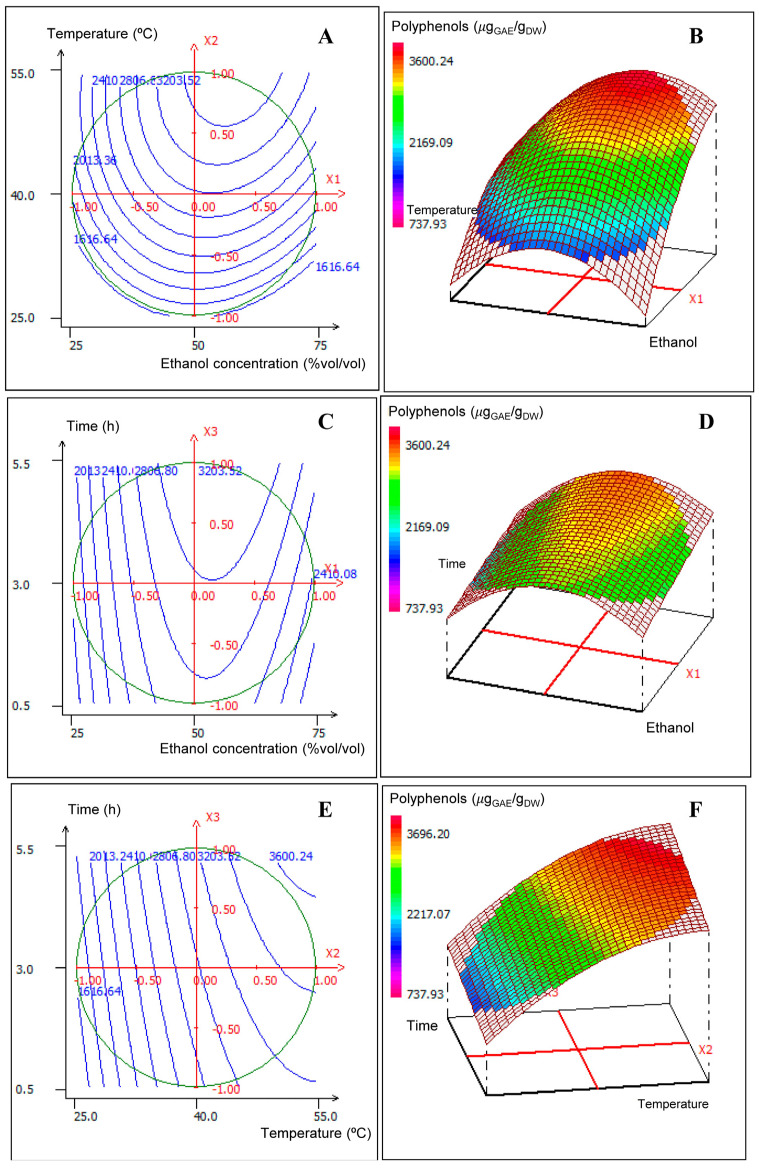
Three-dimensional contour and response surface plots of the variations in the total phenolic content (µg_GAE_/g_DW_) of SE samples. (**A**,**B**) Results at fixed extraction time of 3 h. (**C**,**D**) Results at 40 °C fixed temperature. (**E**,**F**) Results at fixed ethanol concentration of 50%.

The results of the Box–Behnken design experiments of conventional solid–liquid extraction with measured responses of TPC are shown in Figure 1.

Figure 1A,B show that the maximal extraction of polyphenols was achieved in the intervals with 45–60% ethanol and 40–50 °C. Figure 1C,D show that the maximal values of extraction were achieved with 45–55% ethanol and 5 h extraction time. Finally, Figure 1E,F show the maximal values at 40–50 °C and that time did not significantly affect the extraction yield.

**Figure 2:**
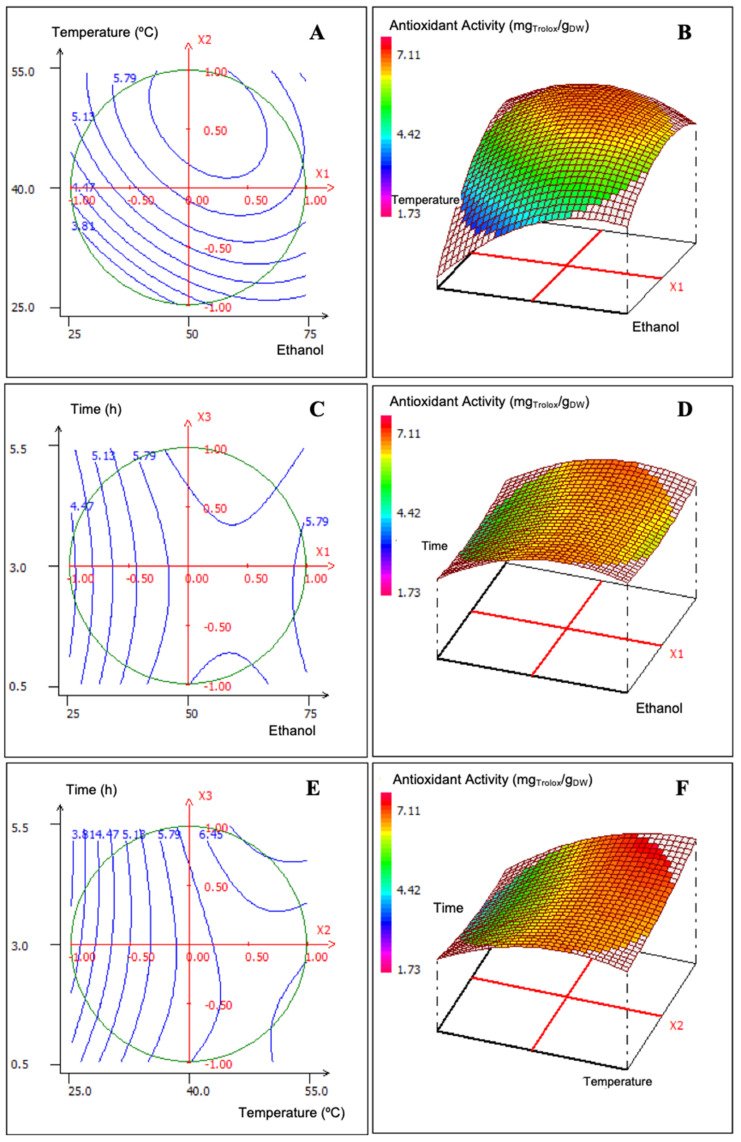
Three-dimensional contour and response surface plots of the variations in the antioxidant activity (mg_Trolox_/g_DW_) of the SE samples. (**A**,**B**) Results at fixed extraction time of 3 h. (**C**,**D**) Results at 40 °C fixed temperature. (**E**,**F**) Results at fixed ethanol concentration of 50%.

Similarly, the response of AA under the designed assays was also studied. The results of these Box–Behnken design experiments are shown in Figure 2.

The results shown in Figure 2 of the AA response plots of the polyphenolic extracts were not significantly different from the TPC responses. Figure 2A,B show the maximal AA of the extracts in the intervals with 45–60% ethanol and 42–50 °C. Figure 2E,F show the maximum extraction in the intervals 40–50 °C and 4–5 h of extraction, and Figure 2C,D show a maximal value with 5 h extraction as well as a secondary value with 1 h extraction. Therefore, the chosen conditions for the conventional SE were 55% ethanol, 50 °C and 5 h. Similarly, other authors reported optimised conditions for SE of phenolic compounds and antioxidant activity from red grape pomaces with ethanol in the concentration range of 30–80% (*v*/*v*) in water at temperatures between 45 °C and 60 °C [32,33,34,35]. Ethanol is the most frequently used solvent in polyphenolic extractions from grape pomace due to its polar character and its recognition as a safe extraction solvent for processing foodstuffs, food components and ingredients [9] by the FAO/WHO Expert Committee on Food Additives [36] and the European Food Safety Authority [37]. Methanol is employed only for experimental studies such as that of Pintac et al. [38], who reported acidified methanol 50% to extract anthocyanins at room temperature from red grape pomaces. In our study, we obtained a total of 250 mL of polyphenolic extract under the optimised SE conditions, and after freeze-drying, 3.4 g of the Es extract was obtained. The TPC of the Es was 368,523 ± 8,400 mg_GAE_/g_DW_, and its AA was 352.58 ± 58.18 mg_Trolox_/g_DW_.

### 3.2. SFE

**Figure 3:**
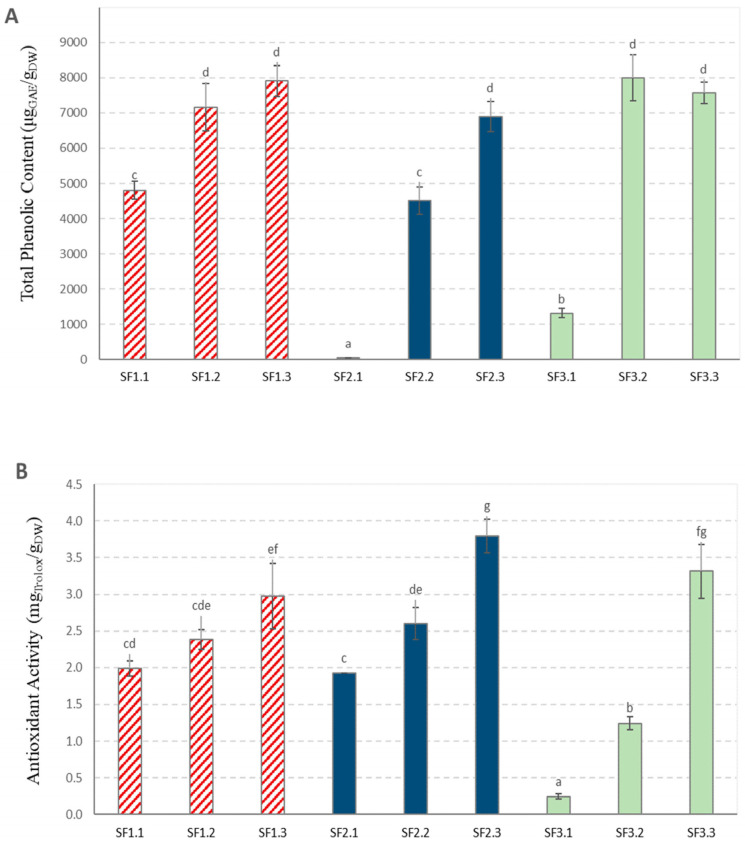
Total phenolic content (**A**) (expressed as µg of equivalent gallic acid per g of dried sample) and antioxidant activity (**B**) (expressed as mg of equivalent Trolox per g of dried sample) of SFE samples. Data are expressed as mean values ± standard deviation of triplicate samples. Different letters indicate significantly different values (*p* ≤ 0.05). Samples recovered from the first extractor: SF1.1, SF2.1, SF3.1. Samples recovered from separator A: SF1.2, SF2.2, SF3.2. Samples recovered from separator B: SF1.3, SF2.3, SF3.3. Samples from assay SF1 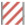. Samples from assay SF2 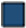. Samples from assay SF3 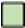.

Figure 3 shows the results of the three SFE assays that were performed under the conditions described in Section 2.6 of Section 2.

The results shown in Figure 3 indicate that, as expected, samples submitted to the whole cycle in the SFE pilot plant, which were recovered from the last separator (B), presented the highest values of both phenolic content and antioxidant activity. Previous studies on SFE from red grape pomaces reported assay pressures in the range from 100 to 400 bar and higher extraction temperatures from 46 to 95 °C [14,17,28,33,39,40,41,42], with the exception of the study of Da Porto et al. [43], who employed 80 bar pressure and 40 °C for SFE from white grape pomace.

The samples we obtained from separator B were named SF1.3, SF2.3 and SF3.3 and were selected for further analysis. It is worth noting that the TPC value (368,523 ± 8400 mg_GAE_/g_DW_) of the SE sample named Es was higher than those of the SFE samples shown in Figure 3. Similarly, some previous studies had also reported that classical SE of grape pomace [43] and grape stalks [33] rendered higher TPC values than SFE. As mentioned before, CO_2_ low polarity does not favour the extraction of polar polyphenols such as red-coloured anthocyanins, and consequently, a number of polyphenols that were present in the SE sample were missing in the SFE samples.

### 3.3. Chemical UPLC-QqQ-MS/MS Analysis of SE and SFE Samples

Chemical analysis by UPLC/QqQ-MS/MS of the Es sample, obtained by SE, identified 38 compounds that were grouped into 7 phenolic families: hydroxycinnamic acids, hydroxybenzoic compounds, flavonols/flavones, flavan 3-ols, procyanidins, stilbenes and anthocyanins. This Es sample was an intense red colour, which was in concordance with its anthocyanin content. In contrast, the colour of the SF1.3, SF2.3 and SF3.3 samples was pale yellow, which revealed the lack of anthocyanins in their composition. Indeed, these samples showed phenolic compounds of the following four families that did not include anthocyanins: hydroxycinnamic acids, hydroxybenzoic acids, flavonols/flavones and stilbenes. It should be noted that red anthocyanins are in their cationic form [44], and their polarity is higher than that of the other phenolic compounds. Although 10% ethanol was included as a co-solvent in the SFE to increase the eluent polarity, anthocyanins were in their red cationic state and were not extracted in the SFE samples. Table 4 shows the total 38 phenolic compounds that were identified in the dried extracts.

Table 5 shows that the Es extract contained 38 phenolic compounds and that the most abundant phenolic family was that of anthocyanins (20,700 ± 521 ng/mg), which accounted for 59% of its phenolic content. The Es anthocyanin content was in the range of 22,600–42,900 ng/mg previously reported for Graciano grape skin extracts [18] and was slightly higher than the reported value (12,225 ± 769 ng/mg) for Graciano dried skins after fermentation [45]. Table 4 shows that among anthocyanins, malvidin stands out as the most abundant anthocyanin in the Es extract. Malvidin accounted for 52% of the total anthocyanin content, and this result is in agreement with reports on the composition of Graciano wines, which are characterised by a high malvidin content that can reach around 65% of the total anthocyanins [30]. The second position in abundance among the Es anthocyanins was delphinidin (Table 4), which is in agreement with the reported composition of Graciano pomace skins, which also showed malvidin as the most abundant anthocyanin, followed by delphinidin [45].

After anthocyanins, the most abundant phenolic families in the Es extract were flavonols/flavones (4247 ± 236 ng/mg), procyanidins (4274 ± 81 ng/mg) and flavan 3-ols (2740 ± 56 ng/mg), followed by lower concentrations of phenolic acids and stilbenes (Table 5). According to reported values of non-coloured phenolic compounds in the dried skins, stems and seeds of Graciano grapes, the stems show the highest contents of procyanidins, flavan 3-ols, phenolic acids and stilbenes when compared with Graciano seeds and skins [45]. Therefore, the composition of our Es extract shows that the optimised SE method was efficient not only on the Graciano pomace skins, but also on the woody short stems in the pomace.

Graciano has been reported as a potential source of stilbene compounds [46], and Table 4 shows that resveratrol is present in the Es sample as well as in the SFE samples, although in lower concentrations. Regarding the composition of flavonols, Table 4 shows that myricetin and quercetin were the most abundant flavonols in the composition of the Es extract, and they together accounted for 82% of the total flavonols/flavones in Es. This result is in agreement with the reported abundance of both flavonols in the composition of Graciano wines [30]. Graciano wines have been also reported to contain higher amounts of catechin than epicatechin [30,47], and in contrast, Graciano seeds have been reported to contain similar concentrations of epicatechin and catechin [47]. Table 4 shows that the contents of both flavan 3-ols, catechin and epicatechin, were similar in the Es extract, which indicates that the SE method was efficient at extracting these flavan 3-ols from the seeds.

As mentioned above, CO_2_ low polarity favours the extraction of less-polar polyphenols in SFE, and thus, the SF1.3, SF2.3 and SF3.3 samples were characterised by their content in phenolic acids and flavonols/flavones, which together accounted for 95% of their total phenolic content, whereas the polyphenols of higher polarity—anthocyanins, procyanidins and flavan 3-ols—were missing in their composition. Regarding the families of chalcones and stilbenes, only small amounts of phlorizin and resveratrol, respectively, were present in the composition of the SFE samples. Similarly to our SFE results with 10% ethanol as co-solvent, SFE from red Garnacha pomace with 8% ethanol co-solvent [14] and SFE from Merlot pomace with 17.5% ethanol co-solvent [40] were reported to extract phenolic compounds of families that did not include anthocyanins. In contrast, studies of red grape pomace SFE with 20% ethanol as cosolvent [41,42] reported the extraction of anthocyanins included among the total extracted polyphenols. These results indicate that the concentration of ethanol as co-solvent in the SFE plays a relevant role in the extraction of anthocyanins, which constitute major components of red grape skins and pomace.

SF2.3 showed the highest contents of total polyphenols, hydroxybenzoic acids, flavonols and resveratrol among the SFE samples. It also showed the highest AA among the SFE samples (Figure 3). This sample was obtained under 600 bar of total pressure in the assay and with the starting material of small particle size (200–500 μm of diameter). The lowest values were shown for SF1.3, which was obtained under 340 bar of total pressure in the assay, and SF3.3, which was obtained from extract particles of 1000–1500 μm of diameter and under 340 bar pressure, showed intermediate values for total polyphenols.

### 3.4. Antimicrobial Activity of the SE and SFE Samples

Once the phenolic compositions of the four extracts, Es, SF1.3, SF2.3 and SF3.3, were determined, they were submitted to the antibacterial activity test described in Section 2.8 of Section 2. The four extracts showed bacterial growth inhibitory activity against our collection of intestinal *E. coli* susceptible to antibiotics and multidrug-resistant strains. Table 6 shows the MIC values of each of the selected extracts against two antibiotic-susceptible *E. coli* strains (C7023 and C7067) and two multidrug-resistant *E. coli* strains (C6840 and C7577).

It is worth noting that in all cases, the observed effect was bacteriostatic and not bactericidal, as bacterial growth recovered after removing the polyphenolic extract from the culture broth and subculturing for 48 h. Gram-negative bacteria are characterised by possessing an additional outer membrane that contains lipopolysaccharides and covers the bacterial cell wall; therefore, Gram-negatives can show higher resistance than Gram-positives to antimicrobial agents that need to reach the cell wall and membrane to exert their bactericidal activity. This could be the case of the *E. coli* strains of our study, which were not killed by the polyphenolic extracts, and recovered after removal of the active polyphenols from the culture broth.

Table 6 shows that the most potent inhibitor was Es, which was able to inhibit the growth of the antibiotic-resistant and the susceptible strains in concentrations in the range of 1–2 mg/mL. Extracts obtained by SFE also were able to inhibit bacterial growth in the range of 2–4 mg/mL. It is worth noting that the total phenolic content (Table 5) correlated with the antimicrobial activity, and thus, Es, which showed the highest phenolic content, showed the highest antimicrobial activity as well, followed by SF2.3, the extract second in phenolic content (3620 ng/mg), SF3.3 (3024 ng/mg) and the last, SF1.3 (1111 ng/mg). The relative abundance of the phenolic families in each of the extracts showed no correlation with the antimicrobial activity of the extract, suggesting that the bacterial growth inhibition mechanisms exerted by the polyphenolic extracts are multi-targeted.

Regarding previous studies on the antimicrobial activity of grape pomace SFE samples, Oliveira et al. [40] reported antimicrobial activity against the *E. coli* antibiotic-sensitive model strain ATCC 25922 of an SFE sample from Merlot pomace. Similarly to our results, they obtained the active extract by supercritical CO_2_ extraction with 17.5% ethanol as co-solvent. The components of that Merlot extract were phenolic acids and flavan 3-ols; neither anthocyanins nor flavones were reported in its composition, and the reported inhibition zone of the extract against the antibiotic-sensitive *E. coli* strain was 9–10 mm. To our knowledge, no other study has been reported to date on the activity of SFE samples from grape pomace, seeds or skins on the growth of antibiotic-resistant *E. coli* strains. One SFE sample obtained from grape stalks was reported to contain flavones, terpenoids, fatty acids and derivates [33] and to possess antimicrobial activity against Gram-positive bacteria, but no reference has been found in the bibliography related to grape-derived samples obtained by SFE that showed antimicrobial activity against antibiotic-resistant *E. coli* strains.

Samples extracted with hydroalcoholic solvents by conventional SE from red grape pomace [48] and red grape skins, seeds and stems [49,50] were reported to lack antimicrobial activity against the antibiotic-sensitive *E. coli* ATCC 25922 [48] and one multidrug-resistant *E. coli* strain [49,50]. In contrast, two studies reported the antimicrobial activity of extracts obtained by conventional SE methods from red grape pomace against antibiotic-resistant *E. coli* strains [51,52]. Sanhueza et al. [51] obtained one extract from red grape pomace by conventional SE and a second liquid–liquid extraction. The major components of the extract were phenolic acids and flavonols (78.8%) and, unlike our SE extract, no anthocyanin was detected in its composition. They reported MIC values of 1.5–3.0 mg/mL against their antibiotic-resistant *E. coli* strains, values in the same range as the MIC values of our extracts. Peixoto et al. [52] reported two hydromethanolic extracts from red grape pomace and from seeds whose MIC values were 20 mg/mL against one antibiotic-resistant *E. coli* strain with a genotype of extended-spectrum β-lactamases. The composition of their conventional SE samples included flavan 3-ols, procyanidins, flavonols, phenolic acids and negligible concentrations of anthocyanins. One SE sample obtained from white grape pomace was also reported to possess antimicrobial activity against another antibiotic-resistant *E. coli* strain with a genotype of extended-spectrum β-lactamases [53]. The major components of their extract were flavan 3-ols, flavonols and procyanidins, and the reported MBC and IC50 (inhibitory concentration that inhibits 50% of microbial growth) values of the extracts against the antibiotic-resistant *E. coli* strain were 15% and 4.65% (*v*/*v*), respectively. In these last two studies [52,53], the reported antimicrobial activities of the SE extracts were at least 10-fold lower than the ones shown in our study.

Regarding the enterohaemorrhagic *E. coli* O157:H7 strain responsible for foodborne infections, some studies of conventional SE samples from red grape pomace [54], white and red grape skins [55] and grape seeds [56] reported antimicrobial activity against this particular *E. coli* strain, and the reported IC50 values and MBC were on the order of 25 mg of solid extract per mL of culture broth [54,56]. Some other reports of antimicrobial activity of conventional SE samples against antibiotic-sensitive *E. coli* strains were reported from grape pomace [57,58,59], grape seeds [60,61] and extracts of grape juice and wines [62,63]. Nevertheless, the reported antibacterial activities vary extensively due to the diversity of the starting raw material, the *Vitis vinifera* variety, the different methods and culture conditions utilised for activity detection and quantification of the phenolic content of the extracts.

The MIC values of the grape pomace extracts of our study, shown in Table 6, are in the range of previously reported values (6.25 mg/mL) of Graciano skin extracts from fresh grapes [25] and are also in accordance with studies on the activity of polyphenols against *E. coli* strains that reported MIC values in the range of 6–18 mg/mL for flavan-3-ols and slightly higher values, in the range of 25–100 mg/mL, for anthocyanins from a variety of vegetable sources [64].

**Figure 4:**
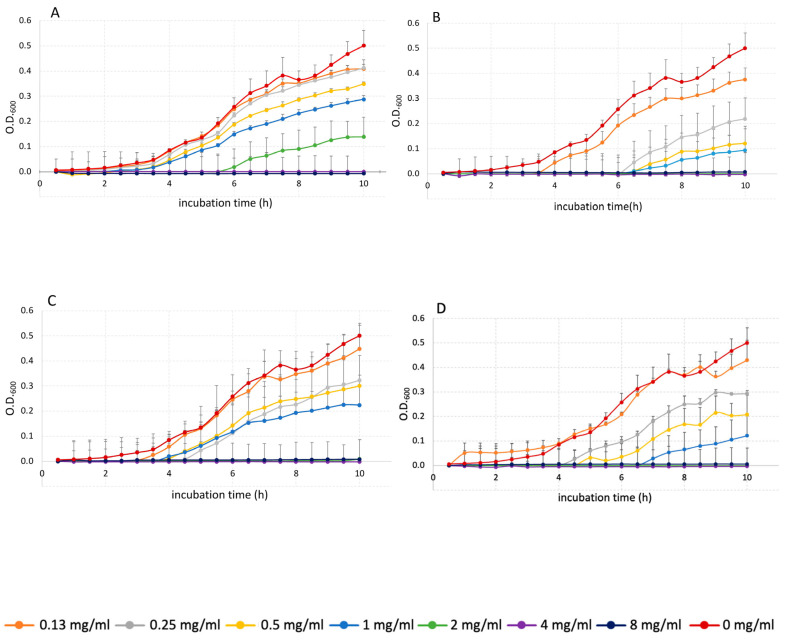
Growth curves of the multidrug-resistant *E. coli* C6840 strain in the presence of increasing concentrations (mg/mL) of each of the polyphenolic extracts: (**A**) SF1.3, (**B**) SF2.3, (**C**) SF3.3 and (**D**) Es. Each dot represents the mean value of triplicates, and bars indicate standard deviations; 0 mg/mL: optical density of control samples of bacterial growth without inhibitors.

Our results with the extracts of the Graciano red grape pomace indicate that this by-product of the winery industry is still a rich source of bioactive compounds and that the most potent inhibitor of bacterial growth, Es, was also the extract with the highest content of all the phenolic families. Es was followed in antibacterial activity and phenolic content by the SF2.3 extract. A direct correlation between both parameters, antibacterial activity and phenolic content, is evidenced by these results. Figure 4 shows some representative growth curves of the antimicrobial activity assays of the four SE and SFE polyphenolic samples against the collection of *E. coli* strains. Additional results of the antibacterial activity of the four SE and SFE extracts against the whole collection of *E. coli* strains are shown in Supplementary Table S2.

In summary, the overall results of this study show that the studied polyphenolic extracts exert an inhibitory effect on the bacterial growth of intestinal *E. coli* strains, both those susceptible to and resistant to antibiotics, and that it is not a bactericidal but a bacteriostatic effect. A higher content of total polyphenols is shown to correlate with higher antibacterial activity of the extracts, whereas the relative abundance of phenolic families shows no correlation with the antibacterial activity, suggesting that the mechanisms by which the inhibition of bacterial growth is exerted by the polyphenolic extracts are multi-targeted. Further research will be required to identify the molecular mechanisms and the specific effectors that trigger bacterial growth inhibition. In addition, this study shows that red grape pomace of *Vitis vinifera* L. cv. Graciano is a rich source of bioactive polyphenolic extracts, and it can become an important feedstock for additives and other upgraded products of biotechnological interest that can help to modulate intestinal microbiota and combat antibiotic-resistant bacteria.

## Figures and Tables

**Table 1:** Design arrangement for optimising conventional SE.

Assay Nr.	Ethanol (%)	Temperature (°C)	Time (h)
1	30	30	3
2	30	30	3
3	70	30	3
4	70	30	3
5	30	50	3
6	30	50	3
7	70	50	3
8	70	50	3
9	30	40	1
10	30	40	1
11	70	40	1
12	70	40	1
13	30	40	5
14	30	40	5
15	70	40	5
16	70	40	5
17	50	30	1
18	50	30	1
19	50	50	1
20	50	50	1
21	50	30	5
22	50	30	5
23	50	50	5
24	50	50	5
25	50	40	3
26	50	40	3
27	50	40	3

**Table 2:** Design arrangement for SFE.

Assay Nr.	Particle Size (µm of Diameter)	Pressure in the First Extraction Vessel (bar)	Pressure in Separator A (bar)	Pressure in Separator B (bar)	Total Pressure in the Assay (bar)
SF1	200–500	150	110	80	340
SF2	200–500	300	200	100	600
SF3	1000–1250	150	110	80	340

**Table 3:** Antibiotic-susceptible and multidrug-resistant *E. coli* strains included in this study.

Strain	Resistance Phenotype	Resistance Genotype	Origin
C7023	Antibiotic susceptible		Cow
C7067	Antibiotic susceptible		Rabbit
C7577	Resistant to: AMP, CAZ, CTX, NAX, TET, SXT	SHV-12	Deer
C6840	Resistant to: AMP, CTX, NAX, TET, SXT	CTX-M-14a	Coatí

AMP (AMP ampicillin), CAZ (ceftazidime), CTX (cefotaxime), NAX (nalidixic acid), TET (tetracycline), SXT (trimethoprim-sulfamethoxazole). SHV-12: genotype of extended-spectrum β-lactamases; CTX-M-14a: genotype of extended-spectrum β-lactamases.

**Table 4:** Contents of phenolic compounds in the extracts (ng/mg of dried extract).

Extract	Es	SF1.3	SF2.3	SF3.3
Name				
Hydroxycinnamic acids				
Caffeic Acid	54.07 ± 1.52 a	6.11 ± 0.45 b	15.02 ± 0.35 c	14.42 ± 0.23 c
Caftaric acid	260.33 ± 54.31 a	nd* b	nd* b	nd* b
Cutaric acid	103.83 ± 1.68 a	nd* b	nd* b	nd* b
Coumaric acid	104.68 ± 2.35 a	57.31 ± 2.02 b	51.19 ± 0.40 c	72.76 ± 0.51 d
Ferulic acid	293.84 ± 7.64 a	9.16 ± 0.54 b	8.93 ± 0.36 b	10.44 ± 2.22 b
Hydroxybenzoic compounds				
Gallic acid	1094.29 ± 13.49 a	375.08 ± 11.48 b	678.81 ± 10.87 c	764.13 ± 10.72 d
Syringic acid	129.42 ± 9.17 a	219.21 ± 2.33 b	304.77 ± 12.39 c	237.01 ± 12.92 b
Protocatechuic acid	21.74 ± 0.21 a	16.94 ± 0.50 b	55.67 ± 1.64 c	44.36 ± 1.03 d
Tyrosol	10.01 ± 0.60 a	5.99 ± 0.53 b	23.18 ± 0.61 c	14.19 ± 0.39 d
4-Hydroxybenzoic acid	9.67 ± 0.51 a	30.69 ± 0.14 b	32.65 ± 0.39 c	31.62 ± 0.74 bc
Vanillic acid	664.81 ± 25.42 a	237.46 ± 7.14 b	499.95 ± 36.78 c	390.28 ± 12.07 d
Flavonols & flavones				
Naringenin	16.80 ± 0.93 a	10.49 ± 0.53 b	11.26 ± 0.12 b	25.25 ± 0.29 c
Isorhamnetin	182.61 ± 3.84 a	67.87 ± 0.20 b	309.04 ± 5.50 c	171.72 ± 1.00 d
Kaempferol	191.85 ± 13.15 a	43.28 ± 1.97 b	426.45 ± 13.15 c	288.24 ± 2.30 d
Myricetin	1301.77 ± 116.99 a	nd* b	9.84 ± 0.51 b	4.34 ± 0.39 b
Quercetin	2362.46 ± 112.48 a	25.27 ± 1.49 b	1108.52 ± 28.07 c	891.46 ± 45.25 d
Laricitrin	56.19 ± 4.78 a	nd* b	3.25 ± 0.11 b	1.87 ± 0.03 b
Syringetin	87.09 ± 3.24 a	1.40 ± 0.08 b	22.11 ± 0.51 c	10.81 ± 0.44 d
Luteolin	12.71 ± 0.17 a	1.38 ± 0.16 b	6.47 ± 0.25 c	8.24 ± 0.35 d
Apigenin	0.74 ± 0.12 a	0.13 ± 0.01 b	0.89 ± 0.11 a	0.97 ± 0.08 a
Astilbin	34.56 ± 1.99 a	nd* b	1.05 ± 0.18 b	1.30 ± 0.10 b
Flavan 3-ols & chalcones				
Catechin	1363.28 ± 45.73 a	nd* b	nd* b	nd* b
Epicatechin	1371.85 ± 34.69 a	nd* b	nd* b	0.33 ± 0.58 b
Phloretin	0.22 ± 0.19 a	nd* a	nd* a	nd* a
Phlorizin	4.39 ± 0.16 a	0.37 ± 0.03 b	0.32 ± 0.04 b	0.36 ± 0.02 b
Procyanidins	4273.93 ± 80.60 a	nd* b	nd* b	nd* b
Stilbenes				
Resveratrol	195.51 ± 2.16 a	2.93 ± 0.27 b	50.93 ± 0.25 c	39.87 ± 1.41 d
Viniferin	29.46 ± 0.70 a	nd* b	nd* b	nd* b
Piceatannol	3.58 ± 0.82 a	nd* b	nd* b	nd* b
Astringin	3.54 ± 0.12 a	nd* b	nd* b	nd* b
Anthocyans				
Petunidin	2702.51 ± 36.89 a	nd* b	nd* b	nd* b
Delphinidin	3597.47 ± 166.09 a	nd* b	nd* b	nd* b
Peonidin	2600.71 ± 31.26 a	nd* b	nd* b	nd* b
Cyanidin	709.51 ± 9.67 a	nd* b	nd* b	nd* b
Malvidin	10,846.30 ± 295.37 a	nd* b	nd* b	nd* b
Pelargonidin	4.35 ± 0.09 a	nd* b	nd* b	nd* b
Vitisin	239.08 ± 1.32 a	nd* b	nd* b	nd* b
Pinotin A	0.26 ± 0.05 a	nd* b	nd* b	nd* b

Data are expressed as mean values ± standard deviation of triplicate samples. Different letters indicate significantly different values (*p* ≤ 0.05). nd*: values below the detection limit.

**Table 5:** Contents of phenolic families in the extracts (ng/mg of dried extract).

Extract	Es	SF1.3	SF2.3	SF3.3
Name				
Hydroxycinnamic acids	816.75 ± 50.53 a	72.58 ± 2.10 b	75.13 ± 0.89 b	97.62 ± 2.31 b
Hydroxybenzoic acids	1929.94 ± 32.71 a	885.38 ± 17.35 b	1595.03 ± 22.17 c	1481.60 ± 31.81 d
Flavonols & flavones	4246.78 ± 235.93 a	149.83 ± 2.18 b	1898.89 ± 9.98 c	1404.20 ± 47.15 d
Flavan 3-ols & chalcones	2739.75 ± 55.83 a	0.37 ± 0.03 b	0.32 ± 0.04 b	0.69 ± 0.58 b
Procyanidins	4273.93 ± 80.60 a	nd* b	nd* b	nd* b
Stilbenes	232.09 ± 2.53 a	2.93 ± 0.27 b	50.93 ± 0.25 c	39.87 ± 1.41 d
Anthocyans	20,700.20 ± 521.71 a	nd* b	nd* b	nd* b
Total phenolic content	34,939.44 ± 807.41 a	1111.11 ± 21.46 b	3620.29 ± 15.99 c	3023.98 ± 51.87 d

Data are expressed as mean values ± standard deviation of triplicate samples. Different letters indicate significantly different values (*p* ≤ 0.05). nd*: values below the detection limit.

**Table 6:** MIC values (mg/mL) of each of the polyphenolic extracts against the studied *E. coli* strains.

	Antibiotic-Resistant Strains		Antibiotic-Susceptible Strains	
Extract	C6840	C7577	C7067	C7023
Es	2	2	2	1
SF1.3	4	4	4	4
SF2.3	2	2	2	2
SF3.3	2	4	4	2

All assays were performed in triplicate.

## Data Availability

The original contributions presented in the study are included in the article/Supplementary Material, further inquiries can be directed to the corresponding author.

## Supplementary Materials

The following supplementary information can be downloaded at: https://www.mdpi.com/article/10.3390/foods14010017/s1, Table S1: (MRM conditions employed for the analysis and quantification of the phenolic compounds determined in the extracts); Table S2: (Growth monitoring of *E. coli* strains in absence (mg/mL = 0) of polyphenolic extracts, and in presence of increasing concentrations of each polyphenolic extract).
