# Supplementary material for: Chemical Profile and Antibacterial Activity of Vitis vinifera L. cv Graciano Pomace Extracts Obtained by Green Supercritical CO2 Extraction Method Against Multidrug-Resistant Escherichia coli Strains
Source: Foods. 2024 Dec 25;14(1):17. doi: 10.3390/foods14010017 (PMC11720172; doi:10.3390/foods14010017)
Supplement: Supplementary file 1 [file foods-14-00017-s001.zip › Foods_2024 Supplementary material _Table S1.pdf]

Table S1: MRM conditions employed for the analysis and quantification of the phenolic compounds determined in the extracts.

m/z Precursor	m/z Quantifier	m/z Qualifier	RT (min)	Compounds	ESI Ion mode	DP	EP	CEP	CE Quantifier	CE Qualifier	CXP Quantifier	CXP Qualifier	Standard to quantify
611.0	287.0	449.0	3.7	cyanidin-3,5-O-diglucoside	+	80	8.0	27.8	50	50	3.0	3.0	cyanidin-3-O-glucoside (Chrysanthemin)
419.0	287.0	213.0	7.1	cyanidin pentoside isomer 1	+	35	7.0	21.7	30	50	2.0	3.0	cyanidin-3-O-glucoside (Chrysanthemin)
419.0	287.0	213.0	9.9	cyanidin pentoside isomer 2	+	35	7.0	21.7	30	50	2.0	3.0	cyanidin-3-O-glucoside (Chrysanthemin)
449.1	287.1	137.1	5.7	cyanidin-3-O-glucoside (Chrysanthemin)	+	51	9.0	22.6	29	73	4.0	4.0	cyanidin-3-O-glucoside (Chrysanthemin)
491.0	287.0	213.0	8.9	cyanidin-3-(6-O-acetyl)-glucoside	+	80	8.0	24.0	50	50	3.0	3.0	cyanidin-3-O-glucoside (Chrysanthemin)
595.0	287.0	213.0	10.0	cyanidin-3-(6-O-coumaroyl) glucoside cis	+	80	8.0	27.3	50	50	3.0	3.0	cyanidin-3-O-glucoside (Chrysanthemin)
595.0	287.0	213.0	10.3	cyanidin-3-(6-O-coumaroyl) glucoside trans	+	80	8.0	27.3	50	50	3.0	3.0	cyanidin-3-O-glucoside (Chrysanthemin)
465.0	303.0	229.0	4.5	delphinidin-3-O-glucoside (Myrtillin)	+	50	7.0	23.1	30	70	2.0	4.0	delphinidin-3-O-glucoside
627.0	303.0	465.0	3.4	delphinidin-3,5-O-diglucoside	+	80	8.0	28.3	50	20	6.0	14.0	delphinidin-3-O-glucoside
435.0	303.0	229.0	6.3	delphinidin pentoside isomer 1	+	35	7.0	22.2	30	70	2.0	4.0	delphinidin-3-O-glucoside
435.0	303.0	229.0	9.1	delphinidin pentoside isomer 2	+	35	7.0	22.2	30	70	2.0	4.0	delphinidin-3-O-glucoside
507.0	303.0	229.0	7.9	delphinidin-3-O-glucoside	+	60	8.0	24.5	50	70	14.0	4.0	delphinidin-3-O-glucoside
611.0	303.0	229.0	9.4	delphinidin-3-(6-O-coumaroyl) glucoside cis	+	80	8.0	27.8	50	70	14.0	4.0	delphinidin-3-O-glucoside
611.0	303.0	229.0	9.8	delphinidin-3-(6-O-coumaroyl) glucoside trans	+	80	8.0	27.8	50	70	14.0	4.0	delphinidin-3-O-glucoside
655.0	331.1	493.0	6.8	malvidin-3,5-O-diglucoside (Malvin)	+	80	8.0	29.2	35	20	6.0	14.0	malvidin-3-O-glucoside (Oenin)
655.0	331.0	493.0	5.8	malvidin-3,7-O-diglucoside	+	80	8.0	29.2	35	20	6.0	6.0	malvidin-3-O-glucoside (Oenin)
463.0	331.1	315.2	7.6	malvidin pentoside isomer 1	+	36	8.0	23.1	35	57	14.0	6.0	malvidin-3-O-glucoside (Oenin)
463.0	331.1	315.2	8.9	malvidin pentoside isomer 2	+	36	8.0	23.1	35	57	14.0	6.0	malvidin-3-O-glucoside (Oenin)
493.2	331.2	315.2	7.4	malvidin-3-O-glucoside (Oenin)	+	56	10.0	24.0	29	57	6.0	6.0	malvidin-3-O-glucoside (Oenin)
781.0	619.0	603.0	4.7	malvidin- 3-O-glucoside-catechin	+	40	10.0	33.2	30	50	6.0	6.0	malvidin-3-O-glucoside (Oenin)
781.0	619.0	603.0	6.3	malvidin- 3-O-glucoside-epicatechin	+	40	10.0	33.2	30	50	6.0	6.0	malvidin-3-O-glucoside (Oenin)
809.0	357.0	341.0	9.6	malvidin-3-O-glucoside-8-ethyl-(epi)catechin	+	40	10.0	34.1	50	50	6.0	6.0	malvidin-3-O-glucoside (Oenin)
603.0	399.0	383.0	8.7	malvidin-3-(6-O-acetyl)-glucoside carboxypyrano	+	50	8.0	27.5	40	60	6.0	6.0	malvidin-3-O-glucoside (Oenin)
559.0	355.0	339.0	9.3	malvidin-3-(6-O-acetyl)-glucoside pyrano	+	50	8.0	26.1	40	60	6.0	6.0	malvidin-3-O-glucoside (Oenin)
707.0	399.0	383.0	10.1	malvidin-3-(6-O-p-coumaroyl) glucoside carboxypyrano	+	50	8.0	30.8	40	60	6.0	6.0	malvidin-3-O-glucoside (Oenin)
663.0	355.0	339.0	10.6	malvidin-3-(6-O-p-coumaroyl) glucoside pyrano	+	50	8.0	29.4	40	60	6.0	6.0	malvidin-3-O-glucoside (Oenin)
625.0	463.0	447.0	11.0	malvidin-3-O-glucoside-4-vinylcatechol (pinotin A) (malvidin 3-O-glucoside caffeic acid)	+	50	8.0	28.2	40	60	6.0	6.0	malvidin-3-O-glucoside (Oenin)
639.0	477.0	461.0	11.7	malvidin-3-O-glucoside-4-vinylguaiacol	+	50	8.0	28.7	40	60	6.0	6.0	malvidin-3-O-glucoside (Oenin)

609.0	447.0	431.0	11.5	malvidin-3-O-glucoside-4-vinylphenol	+	50	8.0	27.7	40	60	6.0	6.0	malvidin-3-O-glucoside (Oenin)
535.0	331.1	315.2	9.9	malvidin-3-(6-O-acetyl)-glucoside	+	60	8.0	25.4	35	50	14.0	6.0	malvidin-3-O-glucoside (Oenin)
667.0	463.0	447.0	11.5	malvidin-3-(6-O-acetyl)-glucoside-4-vinylcatechol	+	50	8.0	29.6	40	60	6.0	6.0	malvidin-3-O-glucoside (Oenin)
681.0	477.0	461.0	11.9	malvidin-3-(6-O-acetyl)-glucoside-4-vinylguaiacol	+	50	8.0	30.0	40	60	6.0	6.0	malvidin-3-O-glucoside (Oenin)
651.0	447.0	431.0	12.0	malvidin-3-(6-O-acetyl)-glucoside-4-vinylphenol	+	50	8.0	29.0	40	60	6.0	6.0	malvidin-3-O-glucoside (Oenin)
851.0	357.0	341.0	10.5	malvidin-3-(6-O-acetyl)-glucoside-8-ethyl-(epi)catechin	+	50	8.0	35.4	40	60	6.0	6.0	malvidin-3-O-glucoside (Oenin)
655.0	331.0	315.0	10.3	malvidin-3-(6-O-caffeoyl) glucoside	+	60	8.0	29.2	35	35	14.0	14.0	malvidin-3-O-glucoside (Oenin)
639.0	331.1	315.2	10.7	malvidin-3-(6-O-coumaroyl) glucoside cis	+	80	8.0	28.7	50	70	14.0	6.0	malvidin-3-O-glucoside (Oenin)
639.0	331.2	315.2	11.1	malvidin-3-(6-O-coumaroyl) glucoside trans	+	80	8.0	28.7	50	70	14.0	6.0	malvidin-3-O-glucoside (Oenin)
771.0	463.0	447.0	12.0	malvidin-3-(6-O-p-coumaroyl) glucoside-4-vinylcatechol	+	50	8.0	32.9	40	60	6.0	6.0	malvidin-3-O-glucoside (Oenin)
785.0	477.0	461.0	12.9	malvidin-3-(6-O-p-coumaroyl) glucoside-4-vinylguaiacol	+	50	8.0	33.3	40	60	6.0	6.0	malvidin-3-O-glucoside (Oenin)
755.0	447.0	431.0	12.7	malvidin-3-(6-O-p-coumaroyl) glucoside-4-vinylphenol	+	50	8.0	32.4	40	60	6.0	6.0	malvidin-3-O-glucoside (Oenin)
927.0	619.0	603.0	8.5	malvidin-3-(6-O-p-coumaroyl) glucoside-(epi)catechin	+	50	8.0	37.8	60	60	6.0	6.0	malvidin-3-O-glucoside (Oenin)
955.0	357.0	341.0	10.2	malvidin-3-(6-O-p-coumaroyl) glucoside-8-ethyl-(epi)catechin	+	50	8.0	38.7	40	60	6.0	6.0	malvidin-3-O-glucoside (Oenin)
943.0	635.0	619.0	10.2	malvidin-3-(6-O-p-coumaroyl) glucoside-(epi)gallocatechin	+	50	8.0	38.3	40	60	6.0	6.0	malvidin-3-O-glucoside (Oenin)
797.0	635.0	619.0	6.8	malvidin-3-O-glucoside-epigallocatechin	+	40	8.0	33.7	30	50	6.0	6.0	malvidin-3-O-glucoside (Oenin)
825.0	357.0	663.0	11.2	malvidin-3-O-glucoside-8-ethyl-(epi)gallocatechin	+	40	10.0	34.6	50	30	6.0	6.0	malvidin-3-O-glucoside (Oenin)
797.0	635.0	619.0	5.5	malvidin-3-O-glucoside-gallocatechin	+	50	8.0	33.7	40	50	6.0	6.0	malvidin-3-O-glucoside (Oenin)
625.0	301.0	463.0	6.5	peonidin-3,5-O-diglucoside	+	80	8.0	28.2	35	20	6.0	14.0	peonidin-3-O-glucoside
433.0	301.0	286.0	7.0	peonidin pentoside isomer 1	+	36	8.0	22.1	35	50	6.0	6.0	peonidin-3-O-glucoside
433.0	301.0	286.0	8.6	peonidin pentoside isomer 2	+	36	8.0	22.1	35	50	6.0	6.0	peonidin-3-O-glucoside
463.2	301.2	286.1	7.0	peonidin-3-O-glucoside	+	56	9.5	23.1	29	47	6.0	4.0	peonidin-3-O-glucoside
505.0	301.0	286.0	9.8	peonidin-3-(6-O-acetyl)-glucoside	+	60	8.0	24.4	35	50	14.0	6.0	peonidin-3-O-glucoside
609.0	301.0	286.0	10.6	peonidin-3-(6-O-coumaroyl) glucoside cis	+	80	8.0	27.7	50	70	14.0	6.0	peonidin-3-O-glucoside
609.0	301.0	286.0	11.0	peonidin-3-(6-O-coumaroyl) glucoside trans	+	80	8.0	27.7	50	70	14.0	6.0	peonidin-3-O-glucoside
641.0	317.0	479.0	5.8	petunidin-3,5-O-diglucoside	+	80	8.0	28.7	50	20	6.0	14.0	petunidin-3-O-glucoside
449.0	317.0	302.0	6.3	petunidin pentoside isomer 1	+	36	8.0	22.6	35	50	14.0	6.0	petunidin-3-O-glucoside
449.0	317.0	302.0	7.7	petunidin pentoside isomer 2	+	36	8.0	22.6	35	50	14.0	6.0	petunidin-3-O-glucoside
479.1	317.0	302.1	6.3	petunidin-3-O-glucoside	+	51	9.0	23.6	29	47	6.0	6.0	petunidin-3-O-glucoside
521.0	317.0	302.0	9.2	petunidin-3-(6-O-acetyl)-glucoside	+	60	8.0	24.9	50	70	14.0	6.0	petunidin-3-O-glucoside
625.0	317.0	302.0	10.1	cis petunidin-3-(6-O-coumaroyl) glucoside	+	80	8.0	28.2	35	50	14.0	6.0	petunidin-3-O-glucoside
625.0	317.0	302.0	10.4	trans petunidin-3-(6-O-coumaroyl) glucoside	+	80	8.0	28.2	35	50	14.0	6.0	petunidin-3-O-glucoside
561.0	399.0	383.0	7.9	vitisin A (malvidin 3-glucoside carboxypyrano)	+	40	7.0	26.2	30	30	6.0	6.0	malvidin-3-O-glucoside (Oenin)

517.0	355.0	339.0	8.5	vitisin B (malvidin-3-glucoside pyrano)	+	35	7.0	24.8	30	30	6.0	6.0	malvidin-3-O-glucoside (Oenin)
178.8	134.2	135.1	5.1	caffeic acid	-	-25	-6.0	-18.3	-40	-18	-2.0	-2.0	caffeic acid
311.0	179.0	135.1	3.3	caftaric acid	-	-60	-5.0	-23.2	-30	-50	-2.0	-2.0	caftaric acid
207.0	132.9	134.9	12.4	caffeic acid ethylester (ethyl caffeate)	-	-65	-10.0	-19.3	-42	-20	-2.0	-2.0	caffeic acid ethylester
181.0	59.0	137.0	4.8	hydrocaffeic acid	-	-55	-9.0	-18.4	-16	-14	-2.0	0.0	caffeic acid
341.0	179.0	135.0	4.1	caffeic acid hexoside A	-	-60	-5.0	-20.0	-30	-50	-2.0	-2.0	caffeic acid
341.0	179.0	135.0	4.6	caffeic acid hexoside B	-	-60	-5.0	-20.0	-30	-50	-2.0	-2.0	caffeic acid
341.0	179.0	135.0	5.1	caffeic acid hexoside C	-	-60	-5.0	-24.3	-30	-50	-2.0	-2.0	caffeic acid
162.9	119.0	92.8	6.7	trans-coumaric acid (trans-4-hydroxycinnamic acid)	-	-20	-10.0	-14.0	-12	-38	-2.0	-2.0	trans-coumaric acid
162.9	119.0	92.8	7.5	cis-coumaric acid (cis-4-hydroxycinnamic acid)	-	-20	-10.0	-17.7	-12	-38	-2.0	-2.0	trans-coumaric acid
295.0	163.0	119.0	4.3	trans-coutaric acid	-	-20	-5.0	-22.6	-30	-50	-2.0	-2.0	trans-coutaric acid
191.0	117.0	145.0	13.8	coumaric acid ethyl ester (ethyl 4-hydroxycinnamate)	-	-50	-10.0	-18.7	-48	-18	-2.0	-2.0	coumaric acid ethyl ester
164.8	147.0	119.1	7.1	hydrocoumaric acid	-	-35	-10.0	-17.8	-20	-20	-2.0	-2.0	trans-coumaric acid
325.0	163.0	119.0	5.1	coumaric acid hexoside A	-	-20	-5.0	-20.0	-30	-50	-2.0	-2.0	trans-coumaric acid
325.0	163.0	119.0	5.4	coumaric acid hexoside B	-	-20	-5.0	-23.7	-30	-50	-2.0	-2.0	trans-coumaric acid
325.0	163.0	119.0	6.0	coumaric acid hexoside C	-	-20	-5.0	-23.7	-30	-50	-2.0	-2.0	trans-coumaric acid
193.0	134.0	178.1	7.9	trans-ferulic acid	-	-25	-8.5	-18.8	-16	-12	-2.0	-4.0	trans-ferulic acid
193.0	134.0	178.1	8.7	cis-ferulic acid	-	-25	-8.5	-18.8	-16	-12	-2.0	-4.0	trans-ferulic acid
325.0	193.0	134.0	5.4	trans-fertaric acid	-	-35	-7.5	-23.7	-18	-34	-3.0	-2.0	trans-fertaric acid
221.0	206.1	133.1	13.9	ferulic acid ethyl ester (ethyl ferulate)	-	-150	-10.0	-19.8	-16	-28	-3.0	-2.0	ferulic acid ethyl ester
168.9	124.9	79.0	1.8	gallic acid	-	-40	-3.5	-17.9	-16	-34	0.0	0.0	gallic acid
331.0	124.9	124.0	2.8	gallic acid hexoside A	-	-60	-5.0	-23.9	-50	-50	-2.0	-2.0	gallic acid
331.0	59.1	169.1	1.9	1-O-Galloyl-b-D-glucose	-	-70	-9.0	-22.0	-48	-26	0.0	-3.0	gallic acid
183.0	124.0	168.9	3.4	methyl gallate	-	-40	-3.5	-18.4	-16	-10	-2.0	-2.0	gallic acid
197.0	124.0	168.9	7.1	ethyl gallate	-	-40	-3.5	-18.9	-16	-16	-2.0	-2.0	gallic acid
196.9	181.9	123.0	5.7	syringic acid	-	-45	-7.0	-18.9	-14	-24	-4.0	-2.0	syringic acid
153.0	109.0	107.6	2.9	protocatechuic acid	-	-35	-10.0	-17.3	-20	-38	-2.0	-2.0	protocatechuic acid
180.9	108.1	152.9	10.0	protocatechuic acid ethyl ester (ethyl 3,4-dihydroxybenzoate)	-	-55	-8.0	-18.4	-32	-32	-2.0	-2.0	protocatechuic acid ethyl ester
137.0	119.1	105.9	4.8	tyrosol	-	-55	-9.0	-16.7	-18	-18	-2.0	-2.0	tyrosol
153.0	123.0	122.4	3.1	hydroxytyrosol	-	-50	-5.5	-17.3	-12	-34	-2.0	0.0	hydroxytyrosol
137.0	93.0	64.9	3.8	4-p-hydroxybenzoic acid	-	-35	-8.5	-16.7	-22	-42	0.0	0.0	p-hydroxybenzoic acid
151.0	106.9	78.9	4.6	4-hydroxyphenylacetic acid	-	-30	-9.0	-17.2	-12	-24	-2.0	-2.0	4-hydroxyphenylacetic acid
167.0	108.0	152.1	5.0	vanillic acid	-	-50	-7.0	-17.8	-24	-12	-2.0	-4.0	vanillic acid

329.0	167.0	123.0	5.0	vanillic acid hexoside A	-	-60	-7.0	-23.8	-30	-50	-4.0	-4.0	vanillic acid
329.0	167.0	123.0	6.5	vanillic acid hexoside B	-	-60	-7.0	-23.8	-30	-50	-4.0	-4.0	vanillic acid
353.0	191.0	173.0	4.7	3-O-Caffeoylquinic acid (chlorogenic acid)	-	-40	-6.0	-24.7	-30	-30	-2.0	-2.0	3-O-Caffeoylquinic acid
353.0	173.0	179.0	5.3	4-O-caffeoylquinic acid (crypto-chlorogenic acid)	-	-40	-6.0	-24.7	-30	-30	-2.0	-2.0	3-O-Caffeoylquinic acid
385.0	223.0	205.0	5.3	sinapinic acid hexoside A	-	-40	-6.0	-25.9	-15	-30	-2.0	-2.0	trans-ferulic acid
385.0	223.0	205.0	6.7	sinapinic acid hexoside B	-	-40	-6.0	-25.9	-15	-30	-2.0	-2.0	trans-ferulic acid
271.0	151.0	119.0	13.0	naringenin	-	-40	-6.0	-21.7	-30	-30	-2.0	-2.0	naringenin
315.0	300.0	151.1	13.5	isorhamnetin	-	-90	-9.5	-24.0	-20	-34	-3.0	-2.0	isorhamnetin
477.0	314.0	271.0	9.9	isorhamnetin-3-O-galactoside (cacticin)	-	-70	-8.0	-29.3	-34	-48	-4.0	-4.0	isorhamnetin-3-O-glucoside
477.0	314.0	271.0	10.1	isorhamnetin-3-O-glucoside (asterin)	-	-70	-8.0	-29.3	-34	-48	-4.0	-4.0	isorhamnetin-3-O-glucoside
491.0	315.0	151.0	10.0	isorhamnetin-3-O-glucuronide	-	-70	-8.5	-29.8	-42	-42	-4.0	-4.0	isorhamnetin-3-O-glucoside
623.1	314.5	271.0	10.0	isorhamnetin-3-O-rutinoside	-	-90	-8.5	-34.7	-40	-78	-4.0	-4.0	isorhamnetin-3-O-glucoside
284.9	93.0	117.0	13.4	kaempferol	-	-95	-9.0	-24.0	-52	-58	0.0	-2.0	kaempferol
447.0	284.0	254.9	9.6	kaempferol-3-O-galactoside (trifolin)	-	-75	-10.5	-28.2	-30	-46	-4.0	-4.0	kaempferol-3-O-glucoside
447.0	284.0	254.9	9.8	kaempferol-3-O-glucoside (astragalin)	-	-75	-10.5	-28.2	-30	-46	-4.0	-4.0	kaempferol-3-O-glucoside
461.0	285.0	151.0	9.8	kaempferol-3-O-glucuronide	-	-60	-10.5	-28.7	-35	-50	-4.0	-4.0	kaempferol-3-O-glucoside
593.0	447.0	240.0	9.8	kaempferol-3-O-rutinoside	-	-60	-10.5	-33.6	-35	-35	-4.0	-4.0	kaempferol-3-O-glucoside
316.9	151.0	137.1	10.1	myricetin	-	-80	-4.5	-18.0	-30	-32	-2.0	-2.0	myricetin
479.1	317.0	272.0	7.4	myricetin-3-O-galactoside	-	-80	-6.0	-29.4	-20	-35	-4.0	-4.0	myricetin-3-O-galactoside
479.0	316.1	271.1	7.5	myricetin-3-O-glucoside	-	-90	-9.5	-22.0	-30	-46	-3.0	-3.0	myricetin-3-O-galactoside
493.0	317.0	179.0	7.5	myricetin-3-O-glucuronide	-	-80	-6.0	-29.9	-35	-35	-4.0	-4.0	myricetin-3-O-galactoside
625.2	479.0	317.0	7.5	myricetin-3-O-rutinoside	-	-80	-8.5	-34.8	-35	-50	-4.0	-4.0	myricetin-3-O-galactoside
301.0	151.0	179.0	11.9	quercetin	-	-55	-10.0	-22.8	-26	-24	-2.2	-2.2	quercetin
463.1	300.0	271.0	8.9	quercetin-3-O-galactoside (hyperin)	-	-70	-6.0	-28.8	-30	-50	-4.0	-4.0	quercetin-3-O-glucoside
463.0	300.0	271.0	9.1	quercetin-3-O-glucoside (isoquercitrin)	-	-80	-9.0	-22.0	-32	-48	-3.0	-3.0	quercetin-3-O-glucoside
477.0	300.9	150.8	9.0	quercetin-3-O-glucuronide (miquelianin)	-	-65	-9.0	-22.0	-28	-50	-3.0	-2.0	quercetin-3-O-glucuronide
609.1	300.0	271.0	9.0	quercetin-3-O-rutinoside	-	-55	-10.0	-34.2	-30	-50	-4.0	-2.2	quercetin-3-O-glucuronide
331.0	151.0	303.0	12.0	laricitrin (3-methylmyricetin)	-	-60	-4.0	-23.9	-50	-35	-2.2	-2.2	syringetin-3-O-glucoside
493.0	330.0	244.0	9.1	laricitrin-3-O-galactoside	-	-40	-10.0	-29.9	-50	-50	-2.2	-2.2	syringetin-3-O-glucoside
493.0	330.0	244.0	9.3	laricitrin-3-O-glucoside	-	-40	-10.0	-29.9	-50	-50	-2.2	-2.2	syringetin-3-O-glucoside
345.0	315.0	330.0	13.5	syringetin (3,5-dimethylmyricetin)	-	-60	-4.0	-24.4	-35	-30	-2.2	-2.2	syringetin-3-O-glucoside
507.1	344.1	257.8	10.0	syringetin-3-O-galactoside	-	-40	-10.0	-30.4	-50	-50	-2.2	-2.2	syringetin-3-O-glucoside
507.1	344.1	257.8	10.1	syringetin-3-O-glucoside	-	-40	-10.0	-30.4	-50	-50	-2.2	-2.2	syringetin-3-O-glucoside

449.0	151.0	285.0	9.2	astilbin (Taxifolin 3-O-rhamnoside)	-	-70	-8.0	-22.0	-32	-28	-2.0	-3.0	Taxifolin 3-O-rhamnoside
449.0	285.0	286.0	9.9	astilbin isomer A	-	-40	-10.0	-28.3	-50	-50	-2.2	-2.2	Taxifolin 3-O-rhamnoside
449.0	285.0	286.0	9.5	astilbin isomer B	-	-40	-10.0	-28.3	-50	-50	-2.2	-2.2	Taxifolin 3-O-rhamnoside
284.9	132.9	132.1	11.8	luteolin	-	-80	-10.0	-18.0	-46	-70	-2.0	-2.0	luteolin
447.1	285.1	65.0	9.3	luteolin-7-O-glucoside	-	-100	-8.5	-20.0	-34	-104	-3.0	0.0	luteolin-7-O-glucoside
268.9	117.0	65.1	13.3	apigenin	-	-85	-12.0	-20.0	-46	-66	-2.0	0.0	apigenin
431.0	268.1	65.2	10.1	apigenin-7-O-glucoside	-	-100	-10.5	-20.0	-38	-116	-3.0	0.0	apigenin-7-O-glucoside
273.0	167.0	123.1	13.4	phloretin	-	-55	-10.0	-21.8	-22	-28	-3.0	-2.0	phloretin
435.1	273.1	167.2	10.7	phlorizin (phloretin-4-glucoside)	-	-70	-9.0	-27.8	-22	-40	-3.0	-3.0	phlorizin
288.9	108.9	122.7	4.6	catechin	-	-40	-10.0	-22.3	-50	-50	-2.2	-2.2	catechin
288.9	108.9	122.7	6.3	epicatechin	-	-40	-10.0	-22.3	-50	-50	-2.2	-2.2	epicatechin
441.0	169.0	289.1	8.8	epicatechin gallate	-	-40	-10.0	-28.0	-50	-50	-2.2	-2.2	epicatechin
305.0	125.0	109.1	2.8	gallocatechin	-	-40	-10.0	-22.9	-50	-50	-2.2	-2.2	gallocatechin
305.0	125.0	109.1	4.0	epigallocatechin	-	-40	-10.0	-22.9	-50	-50	-2.2	-2.2	epicatechin
457.0	169.0	125.0	4.8	epigallocatechin gallate	-	-40	-10.0	-28.6	-50	-50	-2.2	-2.2	epicatechin
577.1	124.9	289.2	4.0	procyanidin dimer B1	-	-70	-10.0	-33.0	-50	-34	-2.2	-4.0	procyanidin dimer B1
577.1	124.9	289.2	5.8	procyanidin dimer B2	-	-70	-10.0	-33.0	-50	-34	-2.2	-4.0	procyanidin dimer B2
577.1	124.9	289.2	7.3	procyanidin dimer B3	-	-70	-10.0	-33.0	-50	-34	-2.2	-4.0	procyanidin dimer B1
577.1	124.9	289.2	5.3	procyanidin dimer B4	-	-70	-10.0	-33.0	-50	-34	-2.2	-4.0	procyanidin dimer B1
577.2	124.9	289.2	9.1	procyanidin dimer B5	-	-70	-10.0	-33.0	-50	-34	-2.2	-4.0	procyanidin dimer B1
575.0	285.0	124.9	9.1	procyanidin dimer A2	-	-70	-10.0	-32.9	-37	-55	-3.0	-3.0	procyanidin dimer A2
865.0	125.0	289.0	7.0	procyanidin trimer C1	-	-70	-10.0	-43.7	-70	-53	-2.0	-3.0	procyanidin trimer C1
227.0	142.9	185.2	10.6	trans-resveratrol	-	-30	-9.5	-20.1	-30	-18	-2.0	-2.0	trans-resveratrol
227.0	142.9	185.2	12.2	cis-resveratrol	-	-40	-10.0	-20.1	-30	-18	-2.2	-2.2	trans-resveratrol
389.0	227.1	184.9	8.4	trans-piceid (trans-resveratrol-glucoside)	-	-40	-10.0	-26.0	-30	-50	-2.2	-2.2	trans-resveratrol-glucoside
389.0	227.1	184.9	10.4	cis-piceid (cis-resveratrol-glucoside)	-	-40	-10.0	-26.0	-30	-50	-2.2	-2.2	trans-resveratrol-glucoside
453.0	359.0	225.0	11.1	epsilon viniferin	-	-40	-10.0	-28.4	-50	-50	-2.2	-2.2	trans-resveratrol
453.0	225.0	347.0	13.4	omega viniferin	-	-40	-10.0	-28.4	-50	-50	-2.2	-2.2	trans-resveratrol
243.0	159.1	200.9	9.3	trans-piceatannol	-	-40	-10.0	-20.6	-50	-50	-2.2	-2.2	trans-resveratrol
243.0	159.1	200.9	10.8	cis-piceatannol	-	-40	-10.0	-20.6	-50	-50	-2.2	-2.2	trans-resveratrol
405.0	243.0	200.9	6.8	trans-astringin	-	-40	-10.0	-26.6	-50	-50	-2.2	-2.2	trans-resveratrol-glucoside
405.0	243.0	200.9	9.4	cis-astringin	-	-40	-10.0	-26.6	-50	-50	-2.2	-2.2	trans-resveratrol-glucoside
357.2	83.1	122.1	13.5	matairesinol	-	-70	-10.0	-24.9	-30	-40	-2.2	-2.2	matairesinol

361.1	165.0	122.0	10.7	secoisolariciresinol	-	-70	-10.0	-25.0	-30	-40	-2.2	-2.2	secoisolariciresinol

m/z: mass-to-charge ratio, RT: Retention Time; ESI: ElectroSpray Ionisation, DP: Declustering Potential; EP: Entrance Potential; CEP: Collision Entrance Potential; CE: Collision Energy; CXP: Collision Exit Potential.
