# Supplementary material for: Chemical Profile and Antibacterial Activity of Vitis vinifera L. cv Graciano Pomace Extracts Obtained by Green Supercritical CO2 Extraction Method Against Multidrug-Resistant Escherichia coli Strains
Source: Foods. 2024 Dec 25;14(1):17. doi: 10.3390/foods14010017 (PMC11720172; doi:10.3390/foods14010017)
Supplement: Supplementary file 1 [file foods-14-00017-s001.zip › Foods_2024 Supplementary material_Table S2.pdf]

Table S2: Growth monitoring of *E. coli* strains in absence (mg/mL = 0) of polyphenolic extracts, and in presence of increasing concentrations of each polyphenolic extract.

E. coli strain: C7577. Polyphenolic extract: Es

mg/mL Es	0		0.13		0.25		0.5		1		2		4		8	
**Incubation time (h)**	**mean**	**deviation**	**mean**	**deviation**	**mean**	**deviation**	**mean**	**deviation**	**mean**	**deviation**	**mean**	**deviation**	**mean**	**deviation**	**mean**	**deviation**
0.5	0.0189	0.0107	0.0000	0.0000	0.0000	0.0000	0.0000	0.0000	0.0000	0.0000	0.0000	0.0000	0.0000	0.0000	0.0000	0.0000
1	0.0248	0.0127	0.0136	0.0617	0.0025	0.0698	-0.0156	0.0617	0.0034	0.1290	-0.0120	0.0968	-0.0047	0.0735	-0.0110	0.0816
1.5	0.0341	0.0173	0.0186	0.0579	-0.0015	0.0561	-0.0315	0.0405	0.0047	0.0695	-0.0060	0.0124	-0.0093	0.0220	-0.0183	0.0856
2	0.0506	0.0253	0.0276	0.0561	-0.0040	0.0454	-0.0090	0.0763	0.0047	0.0649	-0.0065	0.0077	-0.0091	0.0249	-0.0325	0.0508
2.5	0.0802	0.0344	0.0442	0.0514	0.0049	0.0378	-0.0076	0.0539	0.0045	0.0445	-0.0066	0.0055	-0.0092	0.0243	-0.0301	0.0650
3	0.1254	0.0493	0.0726	0.0443	0.0279	0.0386	0.0275	0.0854	0.0084	0.0717	-0.0061	0.0080	-0.0091	0.0231	-0.0415	0.0459
3.5	0.1781	0.0567	0.1091	0.0357	0.0414	0.0141	0.0390	0.0647	0.0094	0.0482	-0.0061	0.0068	-0.0091	0.0241	-0.0462	0.0421
4	0.2293	0.0718	0.1590	0.0420	0.0772	0.0107	0.1120	0.1185	0.0120	0.0702	-0.0059	0.0690	-0.0087	0.0282	-0.0454	0.0428
4.5	0.2850	0.0970	0.1980	0.0383	0.1111	0.0063	0.1118	0.0462	0.0137	0.0265	-0.0056	0.0745	-0.0090	0.0243	-0.0379	0.0539
5	0.3531	0.0930	0.2433	0.0313	0.1552	0.0017	0.1533	0.0290	0.0250	0.0364	-0.0053	0.0499	-0.0092	0.0164	-0.0415	0.0511
5.5	0.3883	0.1087	0.2944	0.0236	0.2015	0.0036	0.2554	0.1323	0.0320	0.0060	-0.0050	0.0245	-0.0091	0.0128	-0.0443	0.0472
6	0.4366	0.1088	0.3458	0.0142	0.2487	0.0191	0.2688	0.0723	0.0400	0.0177	-0.0046	0.0349	-0.0089	0.0158	-0.0441	0.0440
6.5	0.4644	0.0812	0.3856	0.0187	0.2842	0.0210	0.2811	0.0267	0.0500	0.0177	-0.0042	0.0481	-0.0087	0.0126	-0.0414	0.0457
7	0.4996	0.0714	0.4175	0.0115	0.3124	0.0305	0.3184	0.0367	0.0700	0.0125	-0.0040	0.0152	-0.0082	0.0130	-0.0417	0.0479
7.5	0.5428	0.0601	0.4624	0.0098	0.3403	0.0176	0.3338	0.0338	0.0800	0.0290	-0.0034	0.0821	-0.0076	0.0233	-0.0401	0.0444
8	0.5757	0.0782	0.5315	0.0312	0.3653	0.0259	0.3505	0.0015	0.1120	0.0253	-0.0034	0.0336	-0.0073	0.0191	-0.0423	0.0397
8.5	0.5770	0.0636	0.5457	0.0422	0.3943	0.0135	0.3799	0.0065	0.1180	0.0159	-0.0031	0.0385	-0.0073	0.0207	-0.0432	0.0404
9	0.5948	0.0716	0.6003	0.0186	0.4439	0.0312	0.4258	0.0245	0.1200	0.0243	-0.0027	0.0741	-0.0071	0.0194	-0.0416	0.0370
9.5	0.5947	0.0796	0.6156	0.0614	0.4409	0.0237	0.5155	0.1255	0.1260	0.0265	-0.0026	0.0916	-0.0069	0.0174	-0.0400	0.0384
10	0.6079	0.0659	0.6186	0.0487	0.4827	0.0448	0.5803	0.1898	0.1360	0.0029	-0.0025	0.0391	-0.0072	0.0136	-0.0450	0.0370

Assays were performed as indicated in section 2.8 of Materials and Methods, and cell growth was monitored by optical density (O.D.). Extract concentrations (mg/mL) are indicated in red numbers; “mean” values indicate the average of O.D. values of triplicates; “deviation” values indicate the standard deviation of O.D. values of triplicates. Green cells indicate that the strain was able to grow (mainly at lower growth rate than positive control samples without extract). Pink cells indicate that the strain was not growing.

E.coli strain C7577. Polyphenolic extract: SFC33

mg/mL SFC 3.3.	0		0.13		0.25		0.5		1		2		4		8	
**Incubation time (h)**	**mean**	**deviation**	**mean**	**deviation**	**mean**	**deviation**	**mean**	**deviation**	**mean**	**deviation**	**mean**	**deviation**	**mean**	**deviation**	**mean**	**deviation**
0.5	0.0189	0.0107	0.0000	0.0000	0.0000	0.0000	0.0000	0.0000	0.0000	0.0000	0.0000	0.0000	0.0000	0.0000	0.0000	0.0000
1	0.0248	0.0127	0.0053	0.0048	0.0065	0.0059	0.0057	0.0105	0.0109	0.0122	0.0044	0.0320	0.0036	0.1045	-0.0027	0.0681
1.5	0.0341	0.0173	0.0108	0.0045	0.0129	0.0060	0.0105	0.0094	0.0133	0.0099	0.0063	0.0323	0.0069	0.0932	-0.0015	0.0754
2	0.0506	0.0253	0.0208	0.0046	0.0217	0.0050	0.0207	0.0089	0.0214	0.0109	0.0162	0.0315	0.0088	0.1025	-0.0026	0.0837
2.5	0.0802	0.0344	0.0414	0.0051	0.0406	0.0063	0.0414	0.0086	0.0297	0.0093	0.0321	0.0344	0.0118	0.0933	-0.0007	0.0806
3	0.1254	0.0493	0.0735	0.0046	0.0697	0.0054	0.0642	0.0057	0.0460	0.0093	0.0499	0.0323	0.0032	0.0868	-0.0066	0.0851
3.5	0.1781	0.0567	0.1197	0.0050	0.1074	0.0065	0.0973	0.0018	0.0734	0.0087	0.0668	0.0295	0.0033	0.1068	-0.0084	0.0579
4	0.2293	0.0718	0.1628	0.0049	0.1444	0.0077	0.1334	0.0031	0.0963	0.0103	0.0901	0.0345	0.0048	0.1052	-0.0081	0.0592
4.5	0.2850	0.0970	0.2009	0.0048	0.1760	0.0030	0.1676	0.0012	0.1230	0.0139	0.1077	0.0301	0.0060	0.1077	-0.0096	0.0598
5	0.3531	0.0930	0.2454	0.0042	0.2259	0.0082	0.2060	0.0045	0.1817	0.0271	0.1372	0.0331	0.0088	0.0903	-0.0095	0.0768
5.5	0.3883	0.1087	0.3114	0.0012	0.2816	0.0105	0.2497	0.0080	0.2121	0.0128	0.1666	0.0335	0.0098	0.0821	-0.0094	0.0536
6	0.4366	0.1088	0.3620	0.0149	0.3225	0.0045	0.2950	0.0041	0.2490	0.0211	0.2061	0.0274	0.0118	0.0892	-0.0096	0.0617
6.5	0.4644	0.0812	0.3917	0.0101	0.3702	0.0065	0.3255	0.0058	0.2760	0.0162	0.2245	0.0238	0.0140	0.0881	-0.0099	0.0566
7	0.4996	0.0714	0.4540	0.0060	0.4066	0.0056	0.3800	0.0086	0.3137	0.0226	0.2486	0.0233	0.0156	0.0869	-0.0130	0.0720
7.5	0.5428	0.0601	0.4840	0.0102	0.4326	0.0066	0.4059	0.0059	0.3148	0.0204	0.2832	0.0265	0.0177	0.0742	-0.0141	0.0472
8	0.5757	0.0782	0.5178	0.0088	0.4665	0.0043	0.4501	0.0245	0.3489	0.0080	0.3012	0.0360	0.0178	0.0715	-0.0156	0.0714
8.5	0.5770	0.0636	0.5359	0.0130	0.4945	0.0065	0.4616	0.0097	0.3590	0.0194	0.3147	0.0269	0.0191	0.0799	-0.0149	0.0647
9	0.5948	0.0716	0.5545	0.0138	0.5119	0.0077	0.4870	0.0152	0.3735	0.0213	0.3251	0.0256	0.0199	0.0773	-0.0164	0.0696
9.5	0.5947	0.0796	0.5698	0.0198	0.5333	0.0050	0.5004	0.0120	0.3924	0.0192	0.3365	0.0213	0.0185	0.0727	-0.0152	0.0703
10	0.6079	0.0659	0.5784	0.0273	0.5680	0.0086	0.5297	0.0169	0.4400	0.0258	0.3588	0.0200	0.0199	0.0739	-0.0163	0.0823

Assays were performed as indicated in section 2.8 of Materials and Methods, and cell growth was monitored by optical density (O.D.). Extract concentrations (mg/mL) are indicated in red numbers; “mean” values indicate the average of O.D. values of triplicates; “deviation” values indicate the standard deviation of O.D. values of triplicates. Green cells indicate that the strain was able to grow (mainly at lower growth rate than positive control samples without extract). Pink cells indicate that the strain was not growing.

E.coli strain: C7577. Polyphenolic extract: SFC23

mg/mL SFC 2.3.	0		0.13		0.25		0.5		1		2		4		8	
**Incubation time (h)**	**mean**	**deviation**	**mean**	**deviation**	**mean**	**deviation**	**mean**	**deviation**	**mean**	**deviation**	**mean**	**deviation**	**mean**	**deviation**	**mean**	**deviation**
0.5	0.0189	0.0107	0.0000	0.0000	0.0000	0.0000	0.0000	0.0000	0.0000	0.0000	0.0000	0.0000	0.0000	0.0000	0.0000	0.0000
1	0.0248	0.0127	0.0063	0.0023	0.0050	0.0026	0.0042	0.0038	0.0130	0.0061	0.0077	0.0080	0.0161	0.0381	0.0105	0.0727
1.5	0.0341	0.0173	0.0129	0.0020	0.0116	0.0038	0.0093	0.0031	0.0217	0.0059	0.0017	0.0088	0.0121	0.0295	0.0164	0.0675
2	0.0506	0.0253	0.0240	0.0032	0.0213	0.0025	0.0188	0.0041	0.0304	0.0054	0.0028	0.0103	0.0151	0.0359	0.0194	0.0674
2.5	0.0802	0.0344	0.0461	0.0033	0.0412	0.0043	0.0391	0.0063	0.0319	0.0218	0.0046	0.0090	0.0123	0.0642	0.0217	0.0603
3	0.1254	0.0493	0.0774	0.0046	0.0713	0.0034	0.0708	0.0071	0.0735	0.0072	0.0067	0.0158	0.0129	0.0366	0.0235	0.0608
3.5	0.1781	0.0567	0.1267	0.0047	0.1143	0.0041	0.1032	0.0074	0.1033	0.0031	0.0087	0.0126	0.0125	0.0449	0.0247	0.0460
4	0.2293	0.0718	0.1678	0.0026	0.1515	0.0049	0.1353	0.0091	0.1299	0.0061	0.0117	0.0112	0.0132	0.0417	0.0238	0.0589
4.5	0.2850	0.0970	0.2082	0.0026	0.1909	0.0073	0.1691	0.0054	0.1635	0.0092	0.0146	0.0140	0.0173	0.0620	0.0247	0.0520
5	0.3531	0.0930	0.2592	0.0140	0.2396	0.0091	0.2021	0.0038	0.2189	0.0067	0.0187	0.0187	0.0183	0.0366	0.0245	0.0538
5.5	0.3883	0.1087	0.3116	0.0101	0.3009	0.0102	0.2610	0.0070	0.2606	0.0100	0.0211	0.0143	0.0211	0.0383	0.0263	0.0402
6	0.4366	0.1088	0.3453	0.0134	0.3343	0.0108	0.2882	0.0080	0.2846	0.0154	0.0247	0.0128	0.0247	0.0420	0.0270	0.0536
6.5	0.4644	0.0812	0.3903	0.0129	0.3651	0.0109	0.3326	0.0252	0.3104	0.0100	0.0271	0.0162	0.0275	0.0373	0.0253	0.0421
7	0.4996	0.0714	0.4422	0.0050	0.4346	0.0059	0.3816	0.0350	0.3511	0.0171	0.0298	0.0170	0.0301	0.0443	0.0244	0.0488
7.5	0.5428	0.0601	0.4853	0.0056	0.4689	0.0045	0.3963	0.0111	0.3841	0.0170	0.0225	0.0190	0.0319	0.0485	0.0261	0.0580
8	0.5757	0.0782	0.5087	0.0037	0.5115	0.0118	0.4362	0.0208	0.3935	0.0061	0.0235	0.0107	0.0339	0.0498	0.0257	0.0399
8.5	0.5770	0.0636	0.5315	0.0074	0.5315	0.0155	0.4571	0.0050	0.4060	0.0077	0.0236	0.0177	0.0352	0.0585	0.0252	0.0437
9	0.5948	0.0716	0.5480	0.0113	0.5502	0.0175	0.4743	0.0144	0.4262	0.0069	0.0238	0.0033	0.0351	0.0602	0.0247	0.0382
9.5	0.5947	0.0796	0.5640	0.0231	0.5694	0.0207	0.4997	0.0096	0.4410	0.0068	0.0239	0.0062	0.0360	0.0545	0.0243	0.0427
10	0.6079	0.0659	0.5712	0.0054	0.6026	0.0268	0.4996	0.0116	0.4836	0.0119	0.0241	0.0129	0.0402	0.0567	0.0236	0.0453

Assays were performed as indicated in section 2.8 of Materials and Methods, and cell growth was monitored by optical density (O.D.). Extract concentrations (mg/mL) are indicated in red numbers; “mean” values indicate the average of O.D. values of triplicates; “deviation” values indicate the standard deviation of O.D. values of triplicates. Green cells indicate that the strain was able to grow (mainly at lower growth rate than positive control samples without extract). Pink cells indicate that the strain was not growing.

E.coli strain: C7577. Polyphenolic extract: SFC13

mg/mL SFC 1.3.	0		0.13		0.25		0.5		1		2		4		8	
**Incubation time (h)**	**mean**	**deviation**	**mean**	**deviation**	**mean**	**deviation**	**mean**	**deviation**	**mean**	**deviation**	**mean**	**deviation**	**mean**	**deviation**	**mean**	**deviation**
0.5	0.0189	0.0107	0.0000	0.0000	0.0000	0.0000	0.0000	0.0000	0.0000	0.0000	0.0000	0.0000	0.0000	0.0000	0.0000	0.0000
1	0.0248	0.0127	0.0043	0.0017	0.0036	0.0014	0.0041	0.0062	0.0088	0.0130	0.0097	0.0083	0.0078	0.0068	0.0063	0.0209
1.5	0.0341	0.0173	0.0101	0.0020	0.0086	0.0017	0.0090	0.0064	0.0150	0.0134	0.0108	0.0087	0.0091	0.0056	0.0049	0.0117
2	0.0506	0.0253	0.0213	0.0025	0.0188	0.0019	0.0190	0.0068	0.0237	0.0117	0.0164	0.0105	0.0099	0.0042	0.0043	0.0307
2.5	0.0802	0.0344	0.0400	0.0025	0.0362	0.0024	0.0324	0.0066	0.0341	0.0132	0.0240	0.0095	0.0007	0.0024	0.0075	0.0105
3	0.1254	0.0493	0.0701	0.0044	0.0648	0.0026	0.0577	0.0071	0.0585	0.0132	0.0347	0.0058	0.0082	0.0075	0.0080	0.0063
3.5	0.1781	0.0567	0.1153	0.0045	0.1011	0.0058	0.0878	0.0084	0.0880	0.0141	0.0589	0.0057	0.0033	0.0085	0.0083	0.0152
4	0.2293	0.0718	0.1518	0.0078	0.1393	0.0045	0.1200	0.0110	0.1135	0.0145	0.0838	0.0121	0.0055	0.0168	0.0084	0.0096
4.5	0.2850	0.0970	0.1974	0.0145	0.1798	0.0084	0.1563	0.0097	0.1487	0.0231	0.1070	0.0054	0.0066	0.0076	0.0056	0.0273
5	0.3531	0.0930	0.2504	0.0097	0.2370	0.0091	0.2037	0.0148	0.1883	0.0156	0.1529	0.0105	0.0096	0.0158	0.0060	0.0267
5.5	0.3883	0.1087	0.3050	0.0125	0.2986	0.0147	0.2485	0.0162	0.2345	0.0281	0.1860	0.0114	0.0130	0.0145	0.0040	0.0168
6	0.4366	0.1088	0.3438	0.0159	0.3328	0.0136	0.2890	0.0217	0.2629	0.0303	0.2106	0.0192	0.0130	0.0182	0.0039	0.0284
6.5	0.4644	0.0812	0.3863	0.0180	0.3750	0.0132	0.3206	0.0155	0.2975	0.0275	0.2599	0.0360	0.0156	0.0211	0.0027	0.0388
7	0.4996	0.0714	0.4189	0.0183	0.4236	0.0195	0.3634	0.0211	0.3429	0.0331	0.2701	0.0178	0.0121	0.0131	0.0060	0.0173
7.5	0.5428	0.0601	0.4366	0.0158	0.4398	0.0229	0.3718	0.0183	0.3539	0.0347	0.2829	0.0083	0.0122	0.0211	0.0019	0.0202
8	0.5757	0.0782	0.4499	0.0006	0.4588	0.0265	0.3928	0.0162	0.3694	0.0371	0.3190	0.0099	0.0103	0.0415	0.0096	0.0222
8.5	0.5770	0.0636	0.4672	0.0018	0.4744	0.0284	0.4119	0.0186	0.3891	0.0369	0.3305	0.0124	0.0125	0.0247	0.0001	0.0448
9	0.5948	0.0716	0.4809	0.0032	0.4877	0.0375	0.4268	0.0170	0.4011	0.0366	0.3552	0.0089	0.0128	0.0434	0.0072	0.0296
9.5	0.5947	0.0796	0.4938	0.0006	0.5050	0.0281	0.4352	0.0170	0.4117	0.0388	0.3630	0.0219	0.0227	0.0247	0.0013	0.0345
10	0.6079	0.0659	0.5180	0.0093	0.5304	0.0462	0.4729	0.0378	0.4430	0.0491	0.3863	0.0153	0.0230	0.0398	0.0042	0.0345

Assays were performed as indicated in section 2.8 of Materials and Methods, and cell growth was monitored by optical density (O.D.). Extract concentrations (mg/mL) are indicated in red numbers; “mean” values indicate the average of O.D. values of triplicates; “deviation” values indicate the standard deviation of O.D. values of triplicates. Green cells indicate that the strain was able to grow (mainly at lower growth rate than positive control samples without extract). Pink cells indicate that the strain was not growing.

E.coli strain: C7023. Polyphenolic extract: Es

mg/mL Es.	0		0.13		0.25		0.5		1		2		4		8	
**Incubation time (h)**	**mean**	**deviation**	**mean**	**deviation**	**mean**	**deviation**	**mean**	**deviation**	**mean**	**deviation**	**mean**	**deviation**	**mean**	**deviation**	**mean**	**deviation**
0.5	0.0058	0.0006	0.0000	0.0000	0.0000	0.0000	0.0000	0.0000	0.0000	0.0000	0.0000	0.0000	0.0000	0.0000	0.0000	0.0000
1	0.0113	0.0008	0.0147	0.1306	-0.0044	0.0822	0.0315	0.1307	-0.0177	0.0368	-0.0332	0.0449	-0.0688	0.0379	-0.0022	0.0351
1.5	0.0214	0.0009	0.0131	0.1088	-0.0157	0.0522	0.0373	0.0195	-0.0442	0.0167	-0.0540	0.0413	-0.0286	0.0292	-0.0098	0.0246
2	0.0387	0.0016	0.0207	0.0934	-0.0119	0.0423	0.0286	0.1639	-0.0421	0.0147	-0.0661	0.0252	-0.0246	0.0298	-0.0026	0.0389
2.5	0.0631	0.0022	0.0376	0.0856	0.0085	0.0402	0.0351	0.0278	-0.0398	0.0114	-0.0702	0.0215	-0.0258	0.0245	-0.0032	0.0182
3	0.0955	0.0014	0.0673	0.0867	0.0297	0.0353	0.0307	0.0573	-0.0421	0.0905	-0.0704	0.0101	-0.0219	0.0320	-0.0061	0.0748
3.5	0.1404	0.0035	0.1062	0.0839	0.0729	0.0510	0.0243	0.0585	-0.0405	0.0998	-0.0683	0.0101	-0.0216	0.0323	-0.0055	0.0737
4	0.1961	0.0052	0.1608	0.0891	0.1187	0.0639	0.0201	0.0815	-0.0412	0.0324	-0.0613	0.0177	-0.0198	0.0384	-0.0061	0.0707
4.5	0.2492	0.0102	0.2122	0.0739	0.1247	0.0106	0.0214	0.0362	-0.0279	0.0179	-0.0607	0.0146	-0.0209	0.0277	-0.0053	0.0825
5	0.3228	0.0133	0.2697	0.0715	0.1568	0.0167	0.0157	0.0347	-0.0344	0.0105	-0.0650	0.0880	-0.0201	0.0292	-0.0055	0.0808
5.5	0.3900	0.0190	0.3293	0.0568	0.2204	0.0252	0.0093	0.0530	-0.0313	0.0453	-0.0591	0.0139	-0.0178	0.0266	-0.0051	0.0877
6	0.4361	0.0249	0.3905	0.0458	0.2582	0.0293	0.0587	0.0723	-0.0280	0.0499	-0.0572	0.0909	-0.0203	0.0213	-0.0060	0.0739
6.5	0.4917	0.0452	0.4598	0.0580	0.2938	0.0474	0.0282	0.0463	-0.0202	0.0103	-0.0533	0.0869	-0.0190	0.0215	-0.0061	0.0714
7	0.5279	0.0518	0.5257	0.0466	0.3583	0.0445	0.0187	0.0144	-0.0201	0.0477	-0.0537	0.1008	-0.0198	0.0216	-0.0059	0.0740
7.5	0.5282	0.0491	0.5448	0.0575	0.3738	0.0339	0.0187	0.0172	-0.0154	0.0388	-0.0532	0.0776	-0.0185	0.0221	-0.0061	0.0711
8	0.5282	0.0512	0.5790	0.0511	0.4127	0.0315	0.0559	0.0405	-0.0164	0.0364	-0.0479	0.0608	-0.0178	0.0185	-0.0060	0.0770
8.5	0.5351	0.0481	0.5563	0.0381	0.3800	0.0363	0.0932	0.0464	-0.0148	0.0381	-0.0457	0.1063	-0.0129	0.0187	-0.0056	0.0761
9	0.5551	0.0543	0.5738	0.0505	0.3842	0.0439	0.1120	0.0507	-0.0177	0.0380	-0.0485	0.0927	-0.0119	0.0139	-0.0054	0.0776
9.5	0.5647	0.0407	0.5570	0.0919	0.4111	0.0522	0.1524	0.0611	-0.0136	0.0400	-0.0438	0.1149	-0.0092	0.0164	-0.0055	0.0757
10	0.5768	0.0405	0.5737	0.0756	0.4407	0.0930	0.1661	0.0204	-0.0126	0.0448	-0.0451	0.0860	-0.0074	0.0170	-0.0048	0.0880

Assays were performed as indicated in section 2.8 of Materials and Methods, and cell growth was monitored by optical density (O.D.). Extract concentrations (mg/mL) are indicated in red numbers; “mean” values indicate the average of O.D. values of triplicates; “deviation” values indicate the standard deviation of O.D. values of triplicates. Green cells indicate that the strain was able to grow (mainly at lower growth rate than positive control samples without extract). Pink cells indicate that the strain was not growing.

E.coli strain C7023. Polyphenolic extract: SFC33

mg/mL FSC 3.3.	0		0.13		0.25		0.5		1		2		4		8	
**Incubation time (h)**	**mean**	**deviation**	**mean**	**deviation**	**mean**	**deviation**	**mean**	**deviation**	**mean**	**deviation**	**mean**	**deviation**	**mean**	**deviation**	**mean**	**deviation**
0.5	0.0058	0.0006	0.0000	0.0000	0.0000	0.0000	0.0000	0.0000	0.0000	0.0000	0.0000	0.0000	0.0000	0.0000	0.0000	0.0000
1	0.0113	0.0008	0.0054	0.0048	0.0051	0.0111	0.0061	0.0325	0.0080	0.0099	0.0014	0.0417	0.0030	0.0090	-0.0016	0.0201
1.5	0.0214	0.0009	0.0143	0.0047	0.0121	0.0121	0.0129	0.0322	0.0144	0.0113	0.0020	0.0418	-0.0011	0.0096	-0.0040	0.0083
2	0.0387	0.0016	0.0281	0.0038	0.0254	0.0132	0.0230	0.0322	0.0209	0.0119	0.0027	0.0425	-0.0028	0.0084	-0.0067	0.0152
2.5	0.0631	0.0022	0.0491	0.0033	0.0457	0.0127	0.0372	0.0321	0.0396	0.0132	0.0028	0.0418	-0.0003	0.0048	-0.0085	0.0140
3	0.0955	0.0014	0.0773	0.0025	0.0753	0.0118	0.0604	0.0331	0.0572	0.0147	0.0044	0.0427	0.0053	0.0100	-0.0010	0.0103
3.5	0.1404	0.0035	0.1167	0.0023	0.1126	0.0096	0.0889	0.0332	0.0820	0.0149	0.0060	0.0432	0.0080	0.0085	-0.0117	0.0080
4	0.1961	0.0052	0.1653	0.0030	0.1589	0.0116	0.1244	0.0333	0.1100	0.0178	0.0079	0.0435	0.0136	0.0062	-0.0128	0.0106
4.5	0.2492	0.0102	0.2101	0.0021	0.1914	0.0130	0.1547	0.0301	0.1160	0.0175	0.0096	0.0452	0.0117	0.0061	-0.0144	0.0079
5	0.3228	0.0133	0.2630	0.0018	0.2328	0.0153	0.1913	0.0298	0.1431	0.0176	0.0012	0.0426	0.0172	0.0109	-0.0152	0.0193
5.5	0.3900	0.0190	0.3280	0.0003	0.2848	0.0124	0.2391	0.0308	0.1796	0.0215	0.0014	0.0434	0.0201	0.0054	-0.0173	0.0075
6	0.4361	0.0249	0.3938	0.0045	0.3418	0.0138	0.2877	0.0300	0.2206	0.0224	0.0017	0.0450	0.0307	0.0122	-0.0194	0.0102
6.5	0.4917	0.0452	0.4485	0.0043	0.4082	0.0137	0.3301	0.0366	0.2806	0.0264	0.0019	0.0444	0.0394	0.0054	-0.0223	0.0105
7	0.5279	0.0518	0.4833	0.0034	0.4448	0.0120	0.3614	0.0372	0.3127	0.0285	0.0021	0.0390	0.0221	0.0061	-0.0256	0.0149
7.5	0.5282	0.0491	0.5079	0.0021	0.4572	0.0196	0.3743	0.0367	0.3249	0.0304	0.0020	0.0349	0.0043	0.0148	-0.0299	0.0165
8	0.5282	0.0512	0.5095	0.0037	0.4667	0.0161	0.3781	0.0341	0.3261	0.0263	0.0020	0.0257	0.0401	0.0011	-0.0375	0.0128
8.5	0.5351	0.0481	0.5243	0.0034	0.4487	0.0094	0.3867	0.0123	0.2943	0.0199	0.0018	0.0215	0.0097	0.0046	-0.0415	0.0117
9	0.5551	0.0543	0.5319	0.0042	0.4624	0.0057	0.3905	0.0068	0.2850	0.0241	0.0017	0.0313	0.0120	0.0083	-0.0450	0.0107
9.5	0.5647	0.0407	0.5413	0.0006	0.4704	0.0069	0.4047	0.0056	0.2915	0.0176	0.0015	0.0220	0.0139	0.0127	-0.0495	0.0141
10	0.5768	0.0405	0.5500	0.0043	0.5196	0.0154	0.4134	0.0297	0.3375	0.0219	0.0019	0.0374	0.0127	0.0109	-0.0526	0.0136

Assays were performed as indicated in section 2.8 of Materials and Methods, and cell growth was monitored by optical density (O.D.). Extract concentrations (mg/mL) are indicated in red numbers; “mean” values indicate the average of O.D. values of triplicates; “deviation” values indicate the standard deviation of O.D. values of triplicates. Green cells indicate that the strain was able to grow (mainly at lower growth rate than positive control samples without extract). Pink cells indicate that the strain was not growing.

E.coli strain: C7023. Polyphenolic extract: SFC23

mg/mL FSC 2.3.	0		0.13		0.25		0.5		1		2		4		8	
**Incubation time (h)**	**mean**	**deviation**	**mean**	**deviation**	**mean**	**deviation**	**mean**	**deviation**	**mean**	**deviation**	**mean**	**deviation**	**mean**	**deviation**	**mean**	**deviation**
0.5	0.0058	0.0006	0.0000	0.0000	0.0000	0.0000	0.0000	0.0000	0.0000	0.0000	0.0000	0.0000	0.0000	0.0000	0.0000	0.0000
1	0.0113	0.0008	0.0053	0.0024	0.0045	0.0061	0.0044	0.0142	0.0121	0.0209	0.0045	0.0368	-0.0011	0.0385	-0.0021	0.1168
1.5	0.0214	0.0009	0.0138	0.0019	0.0126	0.0064	0.0114	0.0138	0.0196	0.0197	0.0041	0.0366	-0.0023	0.0383	-0.0049	0.1210
2	0.0387	0.0016	0.0281	0.0019	0.0252	0.0065	0.0235	0.0141	0.0288	0.0184	0.0059	0.0354	-0.0032	0.0386	-0.0078	0.1237
2.5	0.0631	0.0022	0.0501	0.0023	0.0481	0.0066	0.0405	0.0155	0.0470	0.0173	0.0085	0.0335	-0.0040	0.0362	-0.0107	0.1233
3	0.0955	0.0014	0.0790	0.0029	0.0767	0.0069	0.0647	0.0158	0.0694	0.0179	0.0020	0.0329	-0.0040	0.0357	-0.0134	0.1242
3.5	0.1404	0.0035	0.1186	0.0029	0.1162	0.0076	0.0967	0.0153	0.0964	0.0172	0.0036	0.0308	-0.0039	0.0308	-0.0154	0.1226
4	0.1961	0.0052	0.1625	0.0055	0.1610	0.0115	0.1336	0.0148	0.1264	0.0162	0.0055	0.0299	-0.0038	0.0297	-0.0172	0.1305
4.5	0.2492	0.0102	0.2043	0.0052	0.1923	0.0121	0.1698	0.0141	0.1410	0.0102	0.0060	0.0288	-0.0046	0.0284	-0.0203	0.1216
5	0.3228	0.0133	0.2543	0.0064	0.2327	0.0121	0.2138	0.0146	0.1756	0.0072	0.0091	0.0267	-0.0039	0.0203	-0.0241	0.1171
5.5	0.3900	0.0190	0.3154	0.0070	0.2884	0.0099	0.2724	0.0153	0.2174	0.0075	0.0119	0.0249	-0.0032	0.0240	-0.0273	0.1183
6	0.4361	0.0249	0.3750	0.0036	0.3456	0.0098	0.3310	0.0211	0.2614	0.0086	0.0150	0.0226	-0.0025	0.0235	-0.0282	0.1406
6.5	0.4917	0.0452	0.4302	0.0107	0.4163	0.0094	0.3690	0.0225	0.3181	0.0095	0.0179	0.0202	-0.0021	0.0145	-0.0349	0.1023
7	0.5279	0.0518	0.4561	0.0093	0.4441	0.0072	0.3793	0.0212	0.3469	0.0100	0.0199	0.0159	-0.0034	0.0069	-0.0376	0.1228
7.5	0.5282	0.0491	0.4744	0.0167	0.4571	0.0081	0.3842	0.0246	0.3629	0.0119	0.0202	0.0172	-0.0048	0.0018	-0.0446	0.0639
8	0.5282	0.0512	0.4854	0.0090	0.4678	0.0046	0.3884	0.0244	0.3682	0.0105	0.0199	0.0179	-0.0068	0.0066	-0.0447	0.0441
8.5	0.5351	0.0481	0.4985	0.0152	0.4646	0.0099	0.4368	0.0218	0.3419	0.0190	0.0201	0.0138	-0.0057	0.0560	-0.0471	0.1052
9	0.5551	0.0543	0.5117	0.0065	0.4815	0.0080	0.4575	0.0222	0.3484	0.0237	0.0199	0.0109	-0.0102	0.0108	-0.0538	0.0604
9.5	0.5647	0.0407	0.5230	0.0088	0.4890	0.0058	0.4582	0.0249	0.3551	0.0220	0.0200	0.0155	-0.0101	0.0220	-0.0504	0.0964
10	0.5768	0.0405	0.5279	0.0133	0.5279	0.0210	0.4391	0.0231	0.4018	0.0137	0.0196	0.0048	-0.0102	0.0149	-0.0528	0.0728

Assays were performed as indicated in section 2.8 of Materials and Methods, and cell growth was monitored by optical density (O.D.). Extract concentrations (mg/mL) are indicated in red numbers; “mean” values indicate the average of O.D. values of triplicates; “deviation” values indicate the standard deviation of O.D. values of triplicates. Green cells indicate that the strain was able to grow (mainly at lower growth rate than positive control samples without extract). Pink cells indicate that the strain was not growing.

E.coli strain: C7023. Polyphenolic extract: SFC1.3

mg/mL FSC 1.3.	0		0.13		0.25		0.5		1		2		4		8	
**Incubation time (h)**	**mean**	**deviation**	**mean**	**deviation**	**mean**	**deviation**	**mean**	**deviation**	**mean**	**deviation**	**mean**	**deviation**	**mean**	**deviation**	**mean**	**deviation**
0.5	0.0058	0.0006	0.0000	0.0000	0.0000	0.0000	0.0000	0.0000	0.0000	0.0000	0.0000	0.0000	0.0000	0.0000	0.0000	0.0000
1	0.0113	0.0008	0.0046	0.0011	0.0046	0.0012	0.0029	0.0015	0.0008	0.0014	-0.0004	0.0154	-0.0071	0.0173	-0.0043	0.0157
1.5	0.0214	0.0009	0.0129	0.0011	0.0123	0.0012	0.0091	0.0012	0.0065	0.0023	-0.0003	0.0144	-0.0166	0.0161	-0.0074	0.0158
2	0.0387	0.0016	0.0258	0.0006	0.0240	0.0002	0.0191	0.0014	0.0136	0.0010	0.0009	0.0147	-0.0176	0.0157	-0.0105	0.0191
2.5	0.0631	0.0022	0.0491	0.0014	0.0459	0.0016	0.0354	0.0017	0.0277	0.0023	0.0088	0.0111	-0.0187	0.0178	-0.0112	0.0138
3	0.0955	0.0014	0.0785	0.0028	0.0746	0.0024	0.0573	0.0031	0.0490	0.0015	0.0226	0.0094	-0.0079	0.0166	-0.0136	0.0022
3.5	0.1404	0.0035	0.1171	0.0039	0.1135	0.0022	0.0886	0.0029	0.0758	0.0020	0.0406	0.0102	-0.0002	0.0189	-0.0150	0.0134
4	0.1961	0.0052	0.1623	0.0051	0.1602	0.0031	0.1235	0.0073	0.1066	0.0016	0.0653	0.0082	0.0017	0.0191	-0.0150	0.0095
4.5	0.2492	0.0102	0.1964	0.0026	0.1953	0.0049	0.1514	0.0105	0.1272	0.0011	0.0781	0.0125	0.0023	0.0165	-0.0163	0.0212
5	0.3228	0.0133	0.2419	0.0037	0.2416	0.0055	0.1902	0.0152	0.1639	0.0035	0.1029	0.0129	0.0032	0.0196	-0.0172	0.0191
5.5	0.3900	0.0190	0.2990	0.0041	0.2942	0.0080	0.2397	0.0161	0.2026	0.0069	0.1350	0.0151	0.0055	0.0196	-0.0146	0.0072
6	0.4361	0.0249	0.3536	0.0097	0.3458	0.0151	0.2928	0.0207	0.2413	0.0085	0.1696	0.0147	0.0077	0.0202	-0.0169	0.0139
6.5	0.4917	0.0452	0.4107	0.0119	0.4085	0.0244	0.3333	0.0379	0.3005	0.0202	0.2053	0.0173	0.0011	0.0225	-0.0161	0.0203
7	0.5279	0.0518	0.4415	0.0193	0.4398	0.0329	0.3497	0.0302	0.3241	0.0312	0.2227	0.0175	0.0013	0.0219	-0.0144	0.0018
7.5	0.5282	0.0491	0.4573	0.0261	0.4423	0.0312	0.3731	0.0119	0.3374	0.0373	0.2294	0.0188	0.0012	0.0379	-0.0133	0.0121
8	0.5282	0.0512	0.4669	0.0253	0.4577	0.0356	0.3859	0.0032	0.3411	0.0369	0.2324	0.0176	0.0012	0.0242	-0.0138	0.0137
8.5	0.5351	0.0481	0.4608	0.0330	0.4700	0.0369	0.3974	0.0333	0.3272	0.0257	0.2359	0.0171	0.0091	0.0488	-0.0165	0.0282
9	0.5551	0.0543	0.4683	0.0348	0.4841	0.0397	0.4290	0.0051	0.3332	0.0232	0.2379	0.0172	0.0087	0.0306	-0.0169	0.0244
9.5	0.5647	0.0407	0.4707	0.0299	0.4888	0.0392	0.4115	0.0461	0.3388	0.0237	0.2473	0.0176	0.0087	0.0220	-0.0169	0.0275
10	0.5768	0.0405	0.4876	0.0215	0.4959	0.0484	0.4043	0.0421	0.3821	0.0470	0.2560	0.0145	0.0012	0.0120	-0.0190	0.0160

Assays were performed as indicated in section 2.8 of Materials and Methods, and cell growth was monitored by optical density (O.D.). Extract concentrations (mg/mL) are indicated in red numbers; “mean” values indicate the average of O.D. values of triplicates; “deviation” values indicate the standard deviation of O.D. values of triplicates. Green cells indicate that the strain was able to grow (mainly at lower growth rate than positive control samples without extract). Pink cells indicate that the strain was not growing.

E.coli strain: C7067. Polyphenolic extract: Es

mg/mL Es	0		0.13		0.25		0.5		1		2		4		8	
**Incubation time (h)**	**mean**	**deviation**	**mean**	**deviation**	**mean**	**deviation**	**mean**	**deviation**	**mean**	**deviation**	**mean**	**deviation**	**mean**	**deviation**	**mean**	**deviation**
0.5	0.0057	0.0004	0.0000	0.0000	0.0000	0.0000	0.0000	0.0000	0.0000	0.0000	0.0000	0.0000	0.0000	0.0000	0.0000	0.0000
1	0.0122	0.0007	0.0088	0.0210	0.0029	0.0237	0.0230	0.0445	0.0011	0.1519	-0.0288	0.1007	-0.0275	0.1349	-0.0025	0.1079
1.5	0.0262	0.0010	0.0233	0.0221	0.0127	0.0188	0.0072	0.0244	0.0015	0.1712	-0.0372	0.1018	-0.0358	0.0781	-0.0066	0.0918
2	0.0531	0.0020	0.0459	0.0181	0.0305	0.0111	0.0252	0.0235	0.0013	0.1727	-0.0366	0.0835	-0.0355	0.0894	-0.0051	0.0693
2.5	0.0999	0.0038	0.0835	0.0151	0.0617	0.0074	0.0831	0.0482	0.0017	0.1038	-0.0363	0.0752	-0.0367	0.0909	-0.0068	0.0547
3	0.1466	0.0091	0.1349	0.0099	0.0929	0.0121	0.0838	0.0448	0.0017	0.0515	-0.0335	0.0827	-0.0352	0.0755	-0.0083	0.0575
3.5	0.1960	0.0056	0.1755	0.0123	0.1309	0.0134	0.0990	0.0096	0.0017	0.0207	-0.0264	0.0958	-0.0324	0.1018	-0.0036	0.0402
4	0.2597	0.0065	0.2294	0.0119	0.1753	0.0189	0.1572	0.0211	0.0098	0.0033	-0.0237	0.0914	-0.0317	0.0757	-0.0046	0.0204
4.5	0.3282	0.0113	0.2924	0.0121	0.2236	0.0247	0.1875	0.0169	0.0071	0.0097	-0.0226	0.0631	-0.0287	0.0584	-0.0051	0.0292
5	0.3859	0.0090	0.3546	0.0127	0.2712	0.0327	0.2571	0.0346	0.0328	0.0037	-0.0173	0.0572	-0.0255	0.0550	-0.0031	0.0373
5.5	0.4384	0.0152	0.4008	0.0167	0.3097	0.0368	0.2907	0.0311	0.0068	0.0454	-0.0191	0.0320	-0.0232	0.0504	-0.0046	0.0337
6	0.4841	0.0153	0.4391	0.0117	0.3374	0.0417	0.3276	0.0465	0.0471	0.0424	-0.0150	0.0342	-0.0220	0.0660	-0.0022	0.0222
6.5	0.5303	0.0171	0.4771	0.0038	0.3612	0.0621	0.3482	0.0319	0.0965	0.0402	-0.0142	0.0162	-0.0192	0.0638	-0.0019	0.0278
7	0.5687	0.0222	0.5082	0.0198	0.3804	0.0711	0.3693	0.0235	0.0940	0.0365	-0.0106	0.0287	-0.0187	0.0739	-0.0017	0.0323
7.5	0.5911	0.0313	0.5482	0.0240	0.4024	0.1008	0.3891	0.0388	0.0889	0.0382	-0.0068	0.0377	-0.0169	0.0616	0.0097	0.0209
8	0.5976	0.0275	0.6080	0.0500	0.4186	0.1049	0.4049	0.0506	0.1345	0.0162	-0.0062	0.0462	-0.0145	0.0512	0.0022	0.0455
8.5	0.6127	0.0371	0.6118	0.0123	0.4229	0.0987	0.4365	0.0592	0.1262	0.0434	-0.0049	0.0506	-0.0150	0.0567	0.0028	0.0492
9	0.6406	0.0601	0.6062	0.0329	0.4277	0.0901	0.4517	0.0833	0.1689	0.0401	-0.0050	0.0399	-0.0128	0.0618	0.0032	0.0440
9.5	0.6426	0.0455	0.6485	0.0845	0.4282	0.0815	0.4308	0.0562	0.1544	0.0283	-0.0531	0.0283	-0.0099	0.0579	0.0040	0.0287
10	0.6372	0.0490	0.6941	0.0837	0.4799	0.0749	0.4637	0.0452	0.1165	0.0258	-0.0258	0.0325	-0.0112	0.0869	0.0049	0.0342

Assays were performed as indicated in section 2.8 of Materials and Methods, and cell growth was monitored by optical density (O.D.). Extract concentrations (mg/mL) are indicated in red numbers; "mean" values indicate the average of O.D. values of triplicates; "deviation" values indicate the standard deviation of O.D. values of triplicates. Green cells indicate that the strain was able to grow (mainly at lower growth rate than positive control samples without extract). Pink cells indicate that the strain was not growing.

E.coli strain: C7067. Polyphenolic extract: SFC33

mg/mL FSC 3.3.	0		0.13		0.25		0.5		1		2		4		8	
**Incubation time (h)**	**mean**	**deviation**	**mean**	**deviation**	**mean**	**deviation**	**mean**	**deviation**	**mean**	**deviation**	**mean**	**deviation**	**mean**	**deviation**	**mean**	**deviation**
0.5	0.0057	0.0004	0.0000	0.0000	0.0000	0.0000	0.0000	0.0000	0.0000	0.0000	0.0000	0.0000	0.0000	0.0000	0.0000	0.0000
1	0.0122	0.0007	0.0070	0.0022	0.0103	0.0061	0.0074	0.0178	0.0068	0.0450	0.0088	0.0582	-0.0043	0.1904	-0.0308	0.1258
1.5	0.0262	0.0010	0.0207	0.0024	0.0237	0.0070	0.0215	0.0194	0.0118	0.0485	0.0224	0.0635	-0.0132	0.1851	0.0395	0.0721
2	0.0531	0.0020	0.0443	0.0037	0.0462	0.0055	0.0431	0.0220	0.0218	0.0470	0.0307	0.0630	-0.0122	0.1868	0.0097	0.0596
2.5	0.0999	0.0038	0.0822	0.0052	0.0796	0.0066	0.0720	0.0232	0.0379	0.0476	0.0435	0.0648	-0.0083	0.1865	0.0026	0.0565
3	0.1466	0.0091	0.1278	0.0047	0.1141	0.0070	0.1043	0.0225	0.0629	0.0448	0.0620	0.0603	-0.0021	0.1866	-0.0560	0.1446
3.5	0.1960	0.0056	0.1700	0.0036	0.1634	0.0063	0.1422	0.0274	0.1050	0.0467	0.0949	0.0675	0.0021	0.1847	-0.0599	0.1581
4	0.2597	0.0065	0.2209	0.0028	0.2118	0.0091	0.1864	0.0339	0.1358	0.0513	0.1192	0.0651	0.0038	0.1941	-0.0113	0.1471
4.5	0.3282	0.0113	0.2764	0.0048	0.2570	0.0077	0.2312	0.0319	0.1699	0.0530	0.1503	0.0690	0.0047	0.1887	-0.0119	0.1547
5	0.3859	0.0090	0.3346	0.0046	0.3096	0.0131	0.2733	0.0500	0.2110	0.0550	0.1647	0.0651	0.0047	0.1888	-0.0156	0.1407
5.5	0.4384	0.0152	0.3754	0.0065	0.3354	0.0240	0.3147	0.0622	0.2212	0.0617	0.1812	0.0711	0.0038	0.1729	-0.0180	0.1524
6	0.4841	0.0153	0.4215	0.0110	0.3872	0.0250	0.3480	0.0662	0.2441	0.0567	0.1973	0.0703	0.0019	0.1755	-0.0220	0.1651
6.5	0.5303	0.0171	0.4698	0.0147	0.4170	0.0218	0.3812	0.0648	0.2733	0.0526	0.2064	0.0730	-0.0019	0.1760	-0.0284	0.1502
7	0.5687	0.0222	0.5010	0.0090	0.4418	0.0191	0.4100	0.0677	0.2915	0.0620	0.2108	0.0675	-0.0030	0.1705	-0.0349	0.1353
7.5	0.5911	0.0313	0.5259	0.0133	0.4988	0.0163	0.4316	0.0540	0.3312	0.0676	0.2101	0.0756	-0.0049	0.1651	-0.0393	0.1870
8	0.5976	0.0275	0.5597	0.0147	0.5382	0.0148	0.4536	0.0614	0.3470	0.0709	0.2078	0.0798	-0.0093	0.1694	-0.0439	0.1694
8.5	0.6127	0.0371	0.5882	0.0133	0.5687	0.0146	0.4790	0.0535	0.3560	0.0643	0.2026	0.0716	-0.0101	0.1655	-0.0515	0.1693
9	0.6406	0.0601	0.6077	0.0168	0.5782	0.0169	0.4887	0.0637	0.3603	0.0623	0.2040	0.0688	-0.0116	0.1659	-0.0536	0.1219
9.5	0.6426	0.0455	0.6366	0.0238	0.5765	0.0194	0.4986	0.0726	0.3347	0.0673	0.1918	0.0761	-0.0157	0.1290	-0.0556	0.1219
10	0.6372	0.0490	0.6371	0.0164	0.5883	0.0216	0.5115	0.0803	0.3435	0.0611	0.1974	0.0718	-0.0168	0.1167	-0.0542	0.1461

Assays were performed as indicated in section 2.8 of Materials and Methods, and cell growth was monitored by optical density (O.D.). Extract concentrations (mg/mL) are indicated in red numbers; “mean” values indicate the average of O.D. values of triplicates; “deviation” values indicate the standard deviation of O.D. values of triplicates. Green cells indicate that the strain was able to grow (mainly at lower growth rate than positive control samples without extract). Pink cells indicate that the strain was not growing.

E.coli strain: C7067 Polyphenolic extract: SFC23

mg/mL FSC 2.3.	0		0.13		0.25		0.5		1		2		4		8	
**Incubation time (h)**	**mean**	**deviation**	**mean**	**deviation**	**mean**	**deviation**	**mean**	**deviation**	**mean**	**deviation**	**mean**	**deviation**	**mean**	**deviation**	**mean**	**deviation**
0.5	0.0057	0.0004	0.0000	0.0000	0.0000	0.0000	0.0000	0.0000	0.0000	0.0000	0.0000	0.0000	0.0000	0.0000	0.0000	0.0000
1	0.0122	0.0007	0.0085	0.0016	0.0078	0.0065	0.0058	0.0007	0.0132	0.0067	0.0083	0.0102	0.0014	0.0092	0.0038	0.0153
1.5	0.0262	0.0010	0.0224	0.0012	0.0197	0.0082	0.0172	0.0010	0.0226	0.0082	0.0024	0.0144	0.0028	0.0015	0.0090	0.0397
2	0.0531	0.0020	0.0468	0.0002	0.0415	0.0107	0.0395	0.0012	0.0385	0.0061	0.0033	0.0149	0.0052	0.0114	0.0103	0.0169
2.5	0.0999	0.0038	0.0857	0.0020	0.0744	0.0137	0.0750	0.0034	0.0609	0.0069	0.0054	0.0152	0.0065	0.0124	0.0127	0.0326
3	0.1466	0.0091	0.1332	0.0028	0.1096	0.0159	0.1133	0.0027	0.0900	0.0042	0.0073	0.0113	0.0083	0.0165	0.0129	0.0077
3.5	0.1960	0.0056	0.1712	0.0016	0.1646	0.0159	0.1560	0.0021	0.1401	0.0045	0.0113	0.0133	0.0106	0.0068	0.0140	0.0160
4	0.2597	0.0065	0.2242	0.0044	0.2148	0.0142	0.2103	0.0023	0.1822	0.0043	0.0148	0.0125	0.0141	0.0098	0.0167	0.0168
4.5	0.3282	0.0113	0.2837	0.0036	0.2671	0.0173	0.2707	0.0048	0.2223	0.0051	0.0183	0.0154	0.0172	0.0104	0.0184	0.0097
5	0.3859	0.0090	0.3439	0.0046	0.3268	0.0273	0.3337	0.0052	0.2720	0.0091	0.0220	0.0149	0.0198	0.0071	0.0201	0.0132
5.5	0.4384	0.0152	0.3849	0.0075	0.3371	0.0206	0.3854	0.0051	0.2854	0.0079	0.0255	0.0121	0.0242	0.0277	0.0234	0.0172
6	0.4841	0.0153	0.4307	0.0070	0.3886	0.0202	0.4269	0.0089	0.3171	0.0032	0.0282	0.0110	0.0268	0.0164	0.0221	0.0134
6.5	0.5303	0.0171	0.4618	0.0069	0.4287	0.0187	0.4637	0.0069	0.3475	0.0004	0.0311	0.0135	0.0292	0.0164	0.0234	0.0319
7	0.5687	0.0222	0.4937	0.0050	0.4603	0.0211	0.4842	0.0026	0.3819	0.0033	0.0336	0.0201	0.0289	0.0038	0.0153	0.0276
7.5	0.5911	0.0313	0.5322	0.0043	0.5307	0.0314	0.5089	0.0026	0.4266	0.0034	0.0353	0.0131	0.0300	0.0187	0.0090	0.0154
8	0.5976	0.0275	0.5638	0.0112	0.5627	0.0289	0.5187	0.0054	0.4510	0.0082	0.0372	0.0162	0.0273	0.0024	0.0351	0.0154
8.5	0.6127	0.0371	0.5907	0.0072	0.5986	0.0442	0.5277	0.0082	0.4706	0.0119	0.0380	0.0117	0.0281	0.0334	0.0058	0.0175
9	0.6406	0.0601	0.6203	0.0085	0.5920	0.0383	0.5442	0.0051	0.4758	0.0073	0.0378	0.0121	0.0272	0.0092	-0.0054	0.0057
9.5	0.6426	0.0455	0.6334	0.0093	0.5961	0.0389	0.5540	0.0097	0.4812	0.0011	0.0391	0.0159	0.0264	0.0202	-0.0095	0.0098
10	0.6372	0.0490	0.6462	0.0056	0.6081	0.0331	0.5594	0.0135	0.4935	0.0047	0.0393	0.0175	0.0240	0.0124	-0.0092	0.0457

Assays were performed as indicated in section 2.8 of Materials and Methods, and cell growth was monitored by optical density (O.D.). Extract concentrations (mg/mL) are indicated in red numbers; “mean” values indicate the average of O.D. values of triplicates; “deviation” values indicate the standard deviation of O.D. values of triplicates. Green cells indicate that the strain was able to grow (mainly at lower growth rate than positive control samples without extract). Pink cells indicate that the strain was not growing.

E.coli strain: C7067. Polyphenolic extract: SFC1.3

mg/mL FSC 1.3.	0		0.13		0.25		0.5		1		2		4		8	
**Incubation time (h)**	**mean**	**deviation**	**mean**	**deviation**	**mean**	**deviation**	**mean**	**deviation**	**mean**	**deviation**	**mean**	**deviation**	**mean**	**deviation**	**mean**	**deviation**
0.5	0.0057	0.0004	0.0000	0.0000	0.0000	0.0000	0.0000	0.0000	0.0000	0.0000	0.0000	0.0000	0.0000	0.0000	0.0000	0.0000
1	0.0122	0.0007	0.0062	0.0012	0.0025	0.0036	-0.0215	0.0207	-0.0173	0.0237	0.0120	0.1116	-0.0359	0.0336	-0.0627	0.0516
1.5	0.0262	0.0010	0.0181	0.0012	0.0155	0.0047	-0.0128	0.0177	-0.0072	0.0243	0.0115	0.1111	-0.0353	0.0337	-0.0642	0.0519
2	0.0531	0.0020	0.0413	0.0015	0.0402	0.0040	0.0043	0.0133	0.0127	0.0240	0.0102	0.1108	-0.0342	0.0335	-0.0649	0.0541
2.5	0.0999	0.0038	0.0785	0.0028	0.0794	0.0067	0.0317	0.0081	0.0409	0.0258	0.0084	0.1123	-0.0335	0.0335	-0.0662	0.0514
3	0.1466	0.0091	0.1176	0.0023	0.1186	0.0068	0.0623	0.0073	0.0690	0.0265	0.0059	0.1180	-0.0313	0.0331	-0.0667	0.0535
3.5	0.1960	0.0056	0.1677	0.0041	0.1685	0.0049	0.1085	0.0061	0.1227	0.0228	0.0178	0.1207	-0.0273	0.0326	-0.0638	0.0548
4	0.2597	0.0065	0.2232	0.0080	0.2221	0.0058	0.1535	0.0127	0.1627	0.0237	0.0182	0.1190	-0.0245	0.0329	-0.0659	0.0533
4.5	0.3282	0.0113	0.2857	0.0121	0.2834	0.0113	0.1966	0.0239	0.2046	0.0232	0.0566	0.1152	-0.0225	0.0329	-0.0643	0.0547
5	0.3859	0.0090	0.3392	0.0128	0.3383	0.0051	0.2418	0.0317	0.2572	0.0206	0.0938	0.1178	-0.0194	0.0326	-0.0630	0.0557
5.5	0.4384	0.0152	0.3699	0.0123	0.3708	0.0122	0.2695	0.0426	0.2672	0.0232	0.1086	0.1184	-0.0194	0.0324	-0.0601	0.0497
6	0.4841	0.0153	0.4098	0.0034	0.4143	0.0081	0.3094	0.0530	0.3038	0.0234	0.1382	0.1079	-0.0154	0.0312	-0.0597	0.0519
6.5	0.5303	0.0171	0.4468	0.0097	0.4521	0.0044	0.3363	0.0558	0.3376	0.0167	0.1640	0.1097	-0.0142	0.0319	-0.0589	0.0519
7	0.5687	0.0222	0.4712	0.0026	0.4785	0.0038	0.3698	0.0608	0.3690	0.0202	0.1961	0.1015	-0.0129	0.0313	-0.0603	0.0492
7.5	0.5911	0.0313	0.4934	0.0107	0.5145	0.0170	0.4050	0.0601	0.4166	0.0264	0.2055	0.1161	-0.0119	0.0315	-0.0604	0.0561
8	0.5976	0.0275	0.5136	0.0060	0.5353	0.0200	0.4255	0.0727	0.4264	0.0090	0.2212	0.1143	-0.0105	0.0305	-0.0658	0.0570
8.5	0.6127	0.0371	0.5279	0.0004	0.5611	0.0227	0.4321	0.0516	0.4534	0.0214	0.2291	0.1161	-0.0099	0.0309	-0.0620	0.0547
9	0.6406	0.0601	0.5329	0.0115	0.5599	0.0196	0.4619	0.0694	0.4645	0.0124	0.2573	0.1185	-0.0106	0.0299	-0.0626	0.0547
9.5	0.6426	0.0455	0.5394	0.0109	0.5778	0.0275	0.4554	0.0892	0.4681	0.0169	0.2434	0.1030	-0.0107	0.0311	-0.0583	0.0526
10	0.6372	0.0490	0.5425	0.0080	0.5936	0.0246	0.4667	0.0862	0.4755	0.0135	0.2570	0.1095	-0.0089	0.0284	-0.0572	0.0511

Assays were performed as indicated in section 2.8 of Materials and Methods, and cell growth was monitored by optical density (O.D.). Extract concentrations (mg/mL) are indicated in red numbers; “mean” values indicate the average of O.D. values of triplicates; “deviation” values indicate the standard deviation of O.D. values of triplicates. Green cells indicate that the strain was able to grow (mainly at lower growth rate than positive control samples without extract). Pink cells indicate that the strain was not growing.
